# Mechanistic interpretability of fine-tuned protein language models for nanobody thermostability prediction

**DOI:** 10.1093/bioinformatics/btag685

**Published:** 2026-09-19

**Authors:** Taihei Murakami, Yuki Hashidate, Yasuhiro Matsunaga

**Affiliations:** Graduate School of Science and Engineering, Saitama University, Saitama 338-8570, Japan; Research & Development Department, Epsilon Molecular Engineering, Inc, Saitama 338-8570, Japan; Graduate School of Science and Engineering, Saitama University, Saitama 338-8570, Japan; Graduate School of Science and Engineering, Saitama University, Saitama 338-8570, Japan; Life and Medical Science Application Interface Platform Development Unit, RIKEN Center for Computational Science, Kobe, Hyogo 650-0047, Japan

## Abstract

**Motivation:**

While Protein Language Models (PLMs) fine-tuned on biophysical data achieve high predictive accuracy, the physical principles underlying their predictions remain obscure. Deciphering these representations offers a unique opportunity to not only interpret model decisions but also to discover novel biophysical insights governing protein properties. Here, we present a framework using Sparse Autoencoders (SAEs) to extract mechanistic knowledge from PLMs fine-tuned for nanobody thermostability.

**Results:**

We fine-tuned the ESM-2 model on the nanobody thermostability dataset, achieving superior performance compared to significantly larger state-of-the-art models. SAE analysis successfully decomposed the model’s dense embeddings into sparse, interpretable features without loss of predictive accuracy. We characterized these features through both global and local analyses. Global analysis provided an aggregate map of position-dependent feature contributions, whereas local analysis identified specific residue-level patterns, including known determinants such as the VHH-tetrad and critical disulfide bonds, as well as candidate stabilizing residues. Free Energy Perturbation calculations supported the structural plausibility of selected residue-level hypotheses. These results show that SAE-based interpretation can generate testable, structurally grounded hypotheses for rational protein engineering.

**Availability:**

The data and source code of the proposed method are available at GitHub (https://github.com/matsunagalab/paper_nanobody-thermostability-sae) and Zenodo (DOI: 10.5281/zenodo.18012027).

## 1 Introduction

Protein Language Models (PLMs) are deep learning models that treat amino acid sequences as natural language, learning sequence grammar and statistical patterns through large-scale self-supervised training. PLMs generate rich embeddings effective for downstream tasks such as structure prediction and functional annotation (Rives *et al*. 2021, Elnaggar *et al*. 2022, Lin *et al*. 2023, Su *et al*. 2025, Hayes *et al*. 2025). However, the internal representations captured by PLMs remain largely elusive, representing a critical gap in our understanding. Recent studies suggest that PLMs strongly reflect information such as evolutionarily conserved sequence motifs and residue-residue covariation/contacts (Zhang *et al*. 2024). Although PLMs pre-trained on diverse protein sequence datasets like UniRef (Suzek *et al*. 2015) retain broad knowledge, they may not fully capture physicochemical properties such as thermostability.

Interpretability techniques for understanding the prediction mechanisms of deep learning models are particularly important in scientific applications, but simple methods such as attention visualization (Rao *et al*. 2021, Vig *et al*. 2021, Leem* et al*. 2022) cannot sufficiently capture individual features or their contributions to predictive performance. Recently, a research group at Anthropic introduced Sparse Autoencoders (SAEs) (Bricken *et al*. 2023) into the field of large language models as a powerful approach that reconstructs internal dense representations as more interpretable monosemantic features and disentangles “superposition” at the neuron level. This method has since been applied to PLMs (Adams *et al*. 2025, Gujral *et al*. 2025, Simon and Zou 2025), where SAEs were independently applied to pre-trained models, demonstrating that biologically meaningful features can be extracted. However, these studies mainly focused on general features encoded in pre-trained models, and task-specific concepts acquired through fine-tuning to predict specific properties have not yet been sufficiently explored.

Supervised Fine-Tuning (SFT) adapts pre-trained models to specific tasks using labeled data, emphasizing task-specific features to enhance performance (Chu *et al*. 2024, Lafita *et al*. 2024, Schmirler *et al*. 2024, Wang *et al*. 2025). However, this performance gain comes with a trade-off: the model’s decision-making process becomes even more opaque, potentially obscuring which features contribute to predictions. Mechanistically elucidating which statistical patterns PLMs learn from data and how these contribute to predicting physicochemical properties is crucial for evaluating performance, detecting biases, and potentially deepening our understanding of biomolecular physics and chemistry.

Among various biophysical tasks for proteins, thermostability is a critical property directly relevant to functional maintenance. For instance, nanobodies (VHHs) are valued for their small size and high antigen recognition (Muyldermans 2013, Alexander and Leong 2024), but their applications require thermostable designs. Conventional methods for evaluating this important property rely on either low-throughput experiments like differential scanning calorimetry (Akiba *et al*. 2019, Ikeuchi *et al*. 2021) or computationally expensive molecular dynamics (MD) simulations (Bekker *et al*. 2019). Therefore, establishing machine learning methods that can rapidly and accurately predict melting temperature (Tm) from sequence alone is strongly needed (Blaabjerg *et al*. 2023, Li *et al*. 2023). Previous studies have shown that general thermostability prediction does not improve simply through increased pre-training or model size expansion of PLMs, suggesting that fine-tuning of PLMs with labeled data is essential for the thermostability prediction of nanobodies (Lin *et al*. 2023). Notably, NbBench (Zhang and Tsuda 2025), a comprehensive benchmark for nanobody property prediction, has demonstrated that models pre-trained specifically on antibody and nanobody sequences, such as AntiBERTa2 (Barton *et al*. 2024) and VHHBERT (Tsuruta *et al*. 2024), exhibit superior predictive power for the thermostability task compared to general-purpose PLMs, highlighting the importance of domain-specific pre-training or fine-tuning and rigorous benchmarking in this field.

This study aims to mechanistically elucidate the features PLMs acquire when fine-tuned for nanobody thermostability. We fine-tuned a PLM for the nanobody thermostability prediction task and constructed a high-accuracy fine-tuned PLM. We then analyzed its internal representations by applying SAEs to decompose the embedding representations into interpretable sparse features. We successfully extracted features significantly correlated with *T_m_* from the fine-tuned model. Analysis of features correlating with high *T_m_* values revealed that they capture not only known factors like VHH-tetrad residues and disulfide bonds but also previously uncharacterized candidate stabilizing residues, while some features correlating with low *T_m_* values showed activation at surface-exposed framework residues. These results demonstrate that a combination of supervised fine-tuning and SAEs provides an effective computational protocol for mechanistic interpretability. This approach bridges the gap between powerful predictive models and scientific insights by enabling the extraction of task-specific concepts from fine-tuned PLMs.

## 2 Methods

### 2.1 Datasets

For SFT, we utilized the thermo-seq dataset from NbBench (Zhang and Tsuda 2025) as the labeled dataset. NbBench is a comprehensive benchmark for nanobody property prediction and represents the current state-of-the-art curated resource for this task, providing standardized training (522 sequences), validation (95 sequences), and test (147 sequences) splits. Adopting this dataset also enables direct comparison of our model’s performance against the benchmark results reported in NbBench. To characterize the sequence similarity structure across splits, we analyzed the distribution of maximum pairwise sequence identity between split pairs (Fig. S1, available as supplementary data at *Bioinformatics* online).

For SAE training, we used sequence data extracted from INDI (Integrated Nanobody Database for Immunoinformatics) (Deszyński *et al*. 2022). INDI is a dataset integrating over 11 million nanobody sequences from various NGS data. We performed clustering at 70% sequence identity using Linclust (Steinegger and Söding 2018) to remove redundancy from this dataset, then randomly extracted 100 000 sequences for use as the SAE training dataset.

### 2.2 Supervised fine-tuning of protein language model

In this study, we used the pre-trained ESM-2 8M model (consisting of 6 layers with embedding dimension $D_{\mathrm{ESM}-\mathrm{hidden}}=320$) as the base model. We performed SFT on this model for the regression task of predicting *T_m_* values from nanobody amino acid sequences (Fig. 1). The model architecture consists of ESM-2 with an added linear regression head. The input to the linear head is a vector obtained by mean-pooling the embedding along the sequence length $N_{\mathrm{aa}}$ direction from the final layer (layer 6). Special tokens, <cls> and <eos>, were excluded from this calculation. First, as a baseline, we trained only the linear head using Ridge regression while keeping the ESM-2 weights frozen. The regularization parameter of Ridge regression was optimized through cross-validation. Hereafter, we refer to this baseline prediction model as the *pre-trained model*.

**Figure 1:**
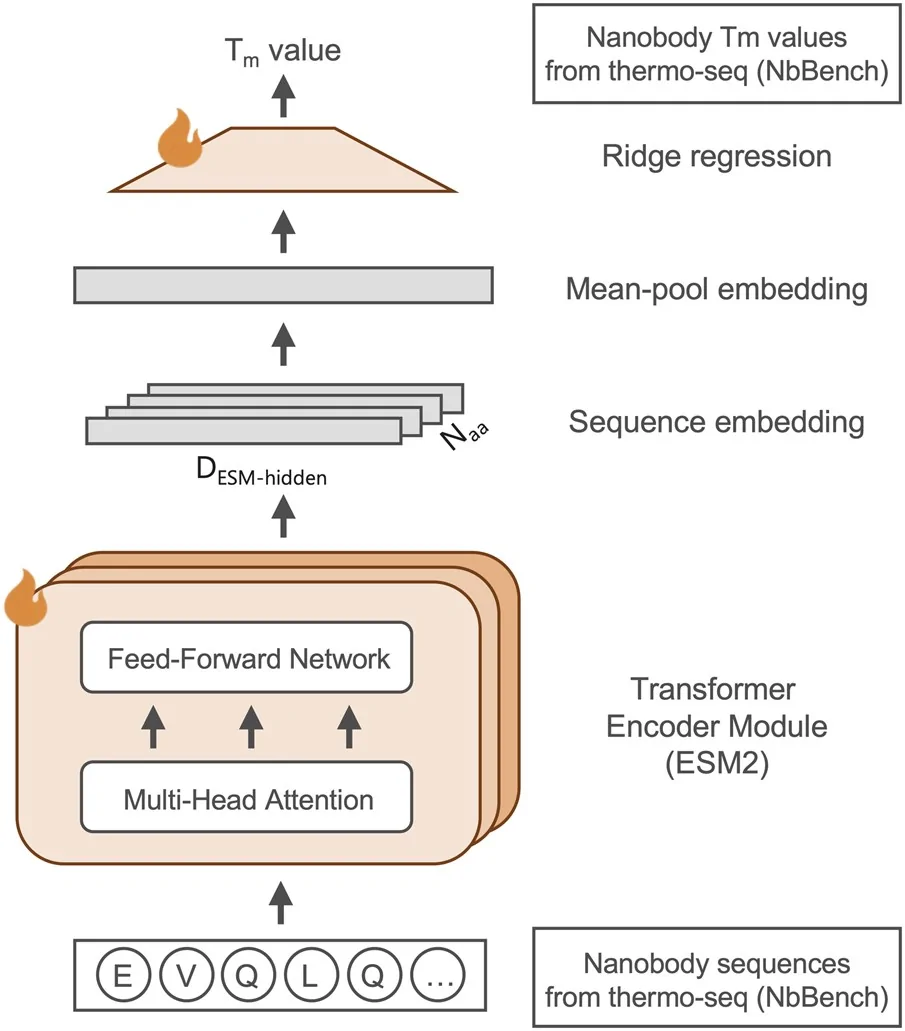
Schematic of the supervised fine-tuning of a protein language model for nanobody thermostability prediction. Architecture of the predictive model. Nanobody sequences are processed by the ESM-2 encoder. The mean-pooled embeddings are fed into a linear head to predict *T_m_*. Both the encoder and linear head weights are updated using the NbBench thermo-seq dataset.

Subsequently, we made both the ESM-2 main body and linear regression head weights updatable. We used the thermo-seq dataset from NbBench (Zhang and Tsuda 2025), which is strictly partitioned into training (522), validation (95), and test (147) samples. For training, we used mean squared error (MSE) as the loss function and optimized the weights of both the ESM-2 encoder and prediction head for 50 epochs. For hyperparameter optimization (learning rate, regularization coefficient, and batch size), we used only the 95 validation samples, completely independent from the test data, performing 100 trials of optimization with Optuna (Akiba *et al*. 2019). Hereafter, we refer to this SFT prediction model as the *SFT model*. Model performance evaluation was performed on the 147 test samples from the thermo-seq dataset, using RMSE, MAE, *R*², Pearson correlation coefficient, and Spearman’s rank correlation coefficient as metrics. To account for the limited size of the test set (*n* = 147), we assessed the robustness of Spearman’s rank correlation for each model (pre-trained and SFT, ESM-2 8M/35M/150M/650M) using bootstrap resampling: 1000 replicates were generated by sampling 147 sequences with replacement from the test set, and the 95% confidence interval (2.5th–97.5th percentile) was computed from the resulting distribution.

### 2.3 Training of sparse autoencoder

To interpret the internal representations, we applied SAEs with the same architecture as Simon and Zou (Fig. 2a). SAEs transform the dense residue embedding representation $x\in R^{D_{\mathrm{ESM}-\mathrm{hidden}}}$ into a higher-dimensional $D_{\mathrm{SAE}-\mathrm{hidden}}\gg D_{\mathrm{ESM}-\mathrm{hidden}}$ but sparse feature vector $z\in R^{D_{\mathrm{SAE}-\mathrm{hidden}}}$, and reconstruct the original embedding representation from these sparse features. Specifically, the SAE encoder transforms x to the sparse latent vector z, and the decoder reconstructs the embedding representation x′ using dictionary vectors $d_{i}\in R^{D_{\mathrm{ESM}-\mathrm{hidden}}}$  $(i=1,\cdots ,D_{\mathrm{SAE}-\mathrm{hidden}}$) with the following equations:


(1) $$z_{i}={\mathrm{ReLU}(W_{e}(x-b_{d})+b_{e})}_{i}$$

(2) $$x^{\prime }=b+\sum_{i=1}^{D_{\mathrm{SAE}-\mathrm{hidden}}}z_{i}d_{i}$$

where $W_{e}$ is a weight matrix, and $b_{d}$, $b_{e}$, b are bias terms. SAE training optimized reconstruction error (MSE) plus L1 norm of latent vector (sparse representation) z as a regularization term. In this study, we processed 100 000 sequences extracted from INDI with the fine-tuned model and used the embedding from the final layer (layer 6) as training targets for the SAE. Although *T_m_* labels were not directly used in the SAE objective, the SAE training targets were embeddings generated by the encoder after supervised fine-tuning on the thermo-seq training set; thus, the learned SAE representation is not independent of the supervised fine-tuning stage. The SAE hidden layer dimension was set to $D_{\mathrm{SAE}-\mathrm{hidden}}=10\;240$ (32× expansion of $D_{\mathrm{ESM}-\mathrm{hidden}}=320$), and training was performed with an L1 norm coefficient of 0.07 after hyperparameter tuning. As a result, the sparsity of z was approximately 1%.

**Figure 2:**
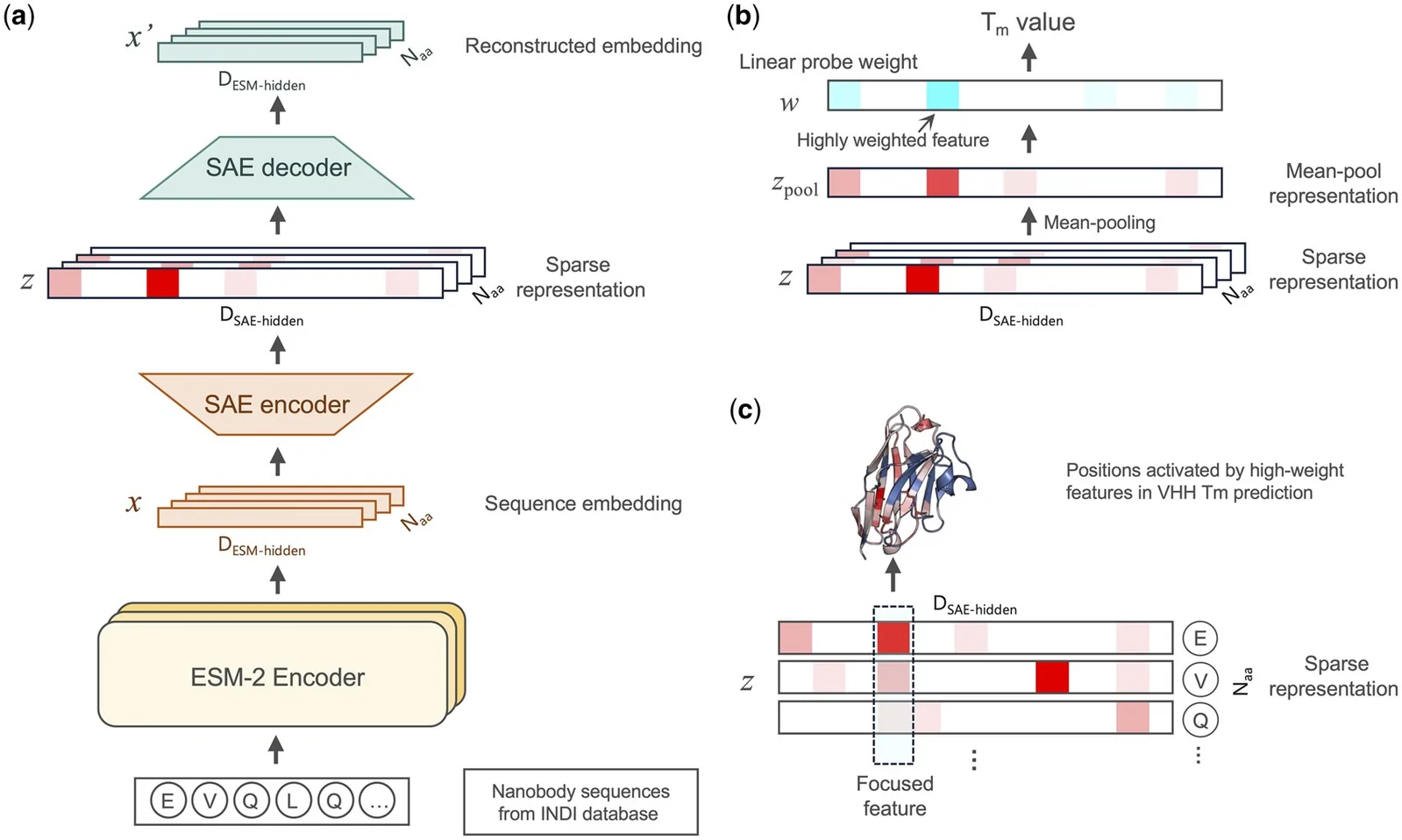
Methodology for interpreting the embedding representation of the fine-tuned model using a Sparse Autoencoder (SAE). (a) Training of the SAE. The embedding representation x, extracted from the final layer of the ESM-2 encoder for unlabeled nanobody sequences, is used to train an SAE. The SAE learns to reconstruct the dense embedding representation x' from a high-dimensional, sparse representation z, effectively decomposing the original embedding into a linear combination of interpretable features. (b) Identifying thermostability-related features. A linear model is trained to predict *T_m_* values directly from the mean-pooled sparse representations $z_{\mathrm{pool}}$. The learned weights *w* of the linear probe indicate the importance of each feature for the prediction task; features with large positive or negative weights are considered highly relevant to thermostability. (c) Visualization and interpretation of features. For a feature identified as important, the specific amino acid residues that cause its activation are identified. These activated residues are then mapped onto the 3D structure of the nanobody to analyze their biophysical and structural significance.

To identify features with large contributions to thermostability, we added a linear head to the latent vector z (Fig. 2b) (Adams *et al*. 2025). In this approach, since the prediction head is linear, features with large absolute weight values in the linear head can be interpreted as important for *T_m_* value prediction. In the calculation of prediction, we first input each sequence from the NbBench thermo-seq dataset to the trained SAE to obtain sparse feature representations. We then mean-pooled the obtained feature representations z along the sequence length $N_{\mathrm{aa}}$ direction, aggregating each sequence into a single vector $z_{\mathrm{pool}}$ of $D_{\mathrm{SAE}-\mathrm{hidden}}=10\;240$ dimensions. The linear head was trained using this vector as input via Ridge regression, after feature-wise scaling of $z_{\mathrm{pool}}$ using MaxAbsScaler (scikit-learn).

To verify whether the SAE sufficiently preserves the predictive information of the SFT model embeddings, we compared *T_m_* prediction accuracy between a linear model using the original embeddings and one using the sparse representations as input. To further verify whether the SFT model’s SAE truly extracts features relevant to thermostability rather than general protein properties, we examined the correlation between individual feature activation strengths and *T_m_* values, comparing SFT-derived and pre-trained model-derived sparse representations. Both models’ predictive calculations used the NbBench thermo-seq test dataset. Subsequently, for NbBench thermo-seq sequences, particularly focusing on sequences with high and low *T_m_* values, we investigated the biophysical significance by visualizing amino acid residues firing in features with large absolute weights in the SAE’s linear head. For structural visualization, amino acid residues firing in features with large absolute weights were visualized onto predicted structures obtained from AlphaFold 3 (Abramson *et al*. 2024).

For interpreting the SAE sparse representation in terms of structure, we visualized how the SAE-extracted features contribute to nanobody thermostability with position-by-position detail along AHo numbering across the entire dataset. All sequences from NbBench thermo-seq dataset were aligned using AHo numbering, with gaps treated as zero. For each feature k with $|w_{k}|\geq 0.05$, we computed the per-position linear contribution as $w_{k}\times z_{k}(p)$, where $w_{k}$ denotes the Ridge weight learned from mean-pooled SAE features and $z_{k}(p)$ is the raw activation of feature k at AHo numbering position p. We then averaged the contributions across the selected features separately for positive ($w_{k}>0$, stabilizing) and negative ($w_{k}<0$, destabilizing) sets to obtain the position-wise profiles. For structural visualization, we mapped these profiles onto AlphaFold 3 structure models of the highest-*T_m_* sequence (for the positive map) and the lowest-*T_m_* sequence (for the negative map) in the dataset.

### 2.4 Free energy perturbation

To quantify the thermodynamic contributions of specific residues identified by the SAE, we performed alchemical free energy perturbation (FEP) calculations. All molecular dynamics simulations for FEP were carried out using NAMD 3 (Phillips *et al*. 2020) with GPU acceleration. We utilized a customized version of the AlaScan plugin (Ramadoss *et al*. 2016) for VMD (Humphrey *et al*. 1996) to support mutations to arbitrary amino acids beyond standard alanine scanning.

The initial structures for FEP were obtained from AlphaFold 3 (Abramson *et al*. 2024) structure predictions of the target nanobody sequences. Each system was prepared using the CHARMM36 protein force field (Best *et al*. 2012). Protein structure files were generated using the psfgen plugin in VMD. Each protein was solvated in a cubic water box of approximately 72 × 72 × 72 Å^3^ using the TIP3P water model (Jorgensen *et al*. 1983), and the system was neutralized and ionized at a physiological salt concentration of 150 mM NaCl. Prior to FEP, the solvated system was energy-minimized followed by a brief equilibration.

Alchemical mutations were performed using a dual-topology approach based on the hybrid residue topology file for CHARMM36. The alchemical transformation was stratified into 20 equally spaced λ windows (Δ*λ* = 0.05) spanning from *λ* = 0 (wild-type) to *λ* =  1 (mutant). A soft-core potential with a van der Waals shift coefficient of 4.0 was employed to avoid end-point singularities. The forward (*λ*: 0 → 1) and backward (*λ*: 1 → 0) transformations each comprised 1 ns of simulation (500 000 steps with a 2-fs timestep), with 200 ps of equilibration per *λ* window before data collection. All simulations were performed in the NPT ensemble at 300 K and 1 atm, using a Langevin thermostat (damping coefficient *γ*  =  1.0 ps^−1^) and Langevin piston barostat. Bond lengths involving hydrogen atoms were constrained using the SHAKE algorithm (Ryckaert *et al*. 1977). Long-range electrostatic interactions were evaluated using the smooth particle mesh Ewald (PME) method (Essmann *et al*. 1995).

The alchemical free-energy change for each WT-to-mutant transformation in the folded nanobody was calculated using the Bennett Acceptance Ratio (BAR) method (Bennett 1976) implemented in the ParseFEP plugin (Liu *et al*. 2012). The mutation-induced change in folding free energy (ΔΔG) was then obtained using a thermodynamic cycle by subtracting the corresponding reference-state contribution. Negative ΔΔG values indicate stabilization and positive values indicate destabilization. Convergence was assessed by monitoring the overlap between adjacent *λ* windows and the hysteresis between the forward and backward free-energy profiles.

## 3 Results

### 3.1 SFT improves prediction and acquires representations reflecting nanobody thermostability

To evaluate the effect of SFT of PLMs on nanobody thermostability prediction, we compared the pre-trained model (model with only the linear head trained) against the SFT model (model where all weights including the ESM-2 portion were fine-tuned on labeled datasets). While the pre-trained model achieved RMSE = 7.702°C, MAE = 6.068°C, *R*² = 0.461, and Spearman = 0.695 on the thermo-seq test set, the SFT model achieved RMSE = 6.749°C, MAE = 4.988°C, *R*² = 0.586, and Spearman = 0.780, demonstrating higher prediction accuracy (Fig. 3a). These results support that, as shown in previous studies (Schmirler *et al*. 2024), fine-tuning the entire model including the PLM improves task-specific prediction performance compared to training only the regression head.

**Figure 3:**
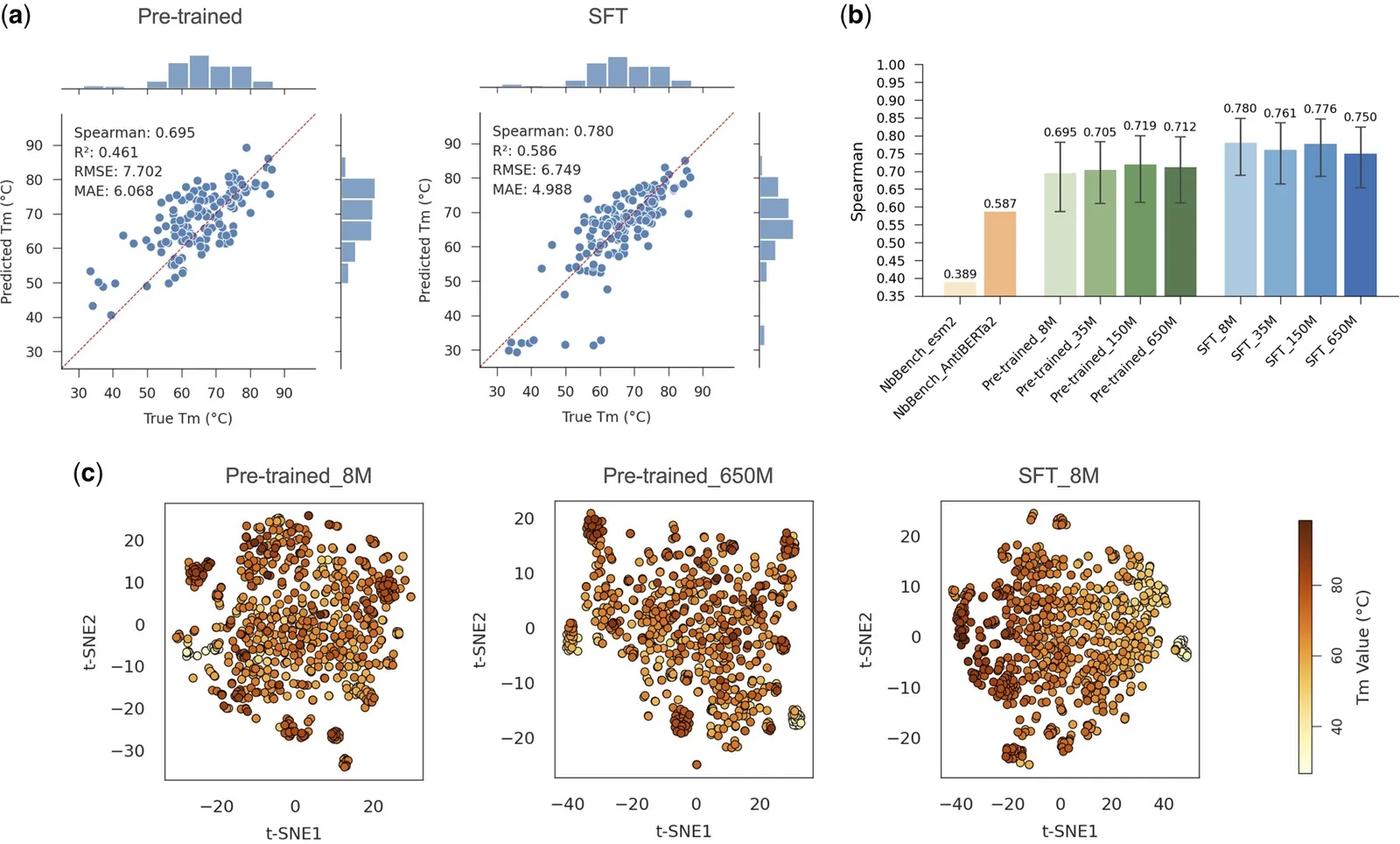
Supervised fine-tuning (SFT) improves thermostability prediction. (a) Comparison of prediction performance on the test set between the pre-trained model (left), where only the final regression head is trained, and the SFT model (right), where the entire model is fine-tuned. Scatter plots show predicted *T_m_* versus true *T_m_*. (b) Benchmarking of model performance. The Spearman’s rank correlation coefficient of our pre-trained and SFT models (based on ESM-2 8M) is compared with the reported performance of baseline models from NbBench (ESM-2 and AntiBERTa2), as well as results for ESM-2 models of increasing size (8M, 35M, 150M, 650M) for both pre-trained and SFT conditions. Error bars indicate 95% confidence intervals obtained by bootstrap resampling of the test set (147 sequences, sampled with replacement, 1000 iterations). (c) t-SNE visualization of the embedding representations from the final layer of the pre-trained 8M (left), pre-trained 650M (center) and SFT 8M (right) ESM-2 encoders. Each point represents a nanobody sequence from the test, validation and training sets, colored by its experimental *T_m_* value.

Furthermore, we compared the performance of our constructed model with the baseline models reported in NbBench (Zhang and Tsuda 2025). The general-purpose ESM-2 model achieved a Spearman correlation of 0.389 on the thermo-seq benchmark, while AntiBERTa2 (Barton *et al*. 2024), a model pre-trained specifically on antibody and nanobody sequences, achieved a higher Spearman correlation of 0.587. Our SFT model (based on ESM-2 8M) achieved Spearman = 0.780 (95% CI: 0.689–0.849, bootstrap), substantially outperforming both baselines (Fig. 3b). This result demonstrates that the fine-tuning approach, despite being based on a general-purpose PLM, achieves state-of-the-art performance for nanobody thermostability prediction, surpassing even domain-specific pre-trained models. Furthermore, to assess whether prediction performance is limited by model size, we scaled up the ESM-2 base model to 35M, 150M, and 650M and evaluated performance for both the pre-trained and SFT models. Within the framework of our method, increasing model size did not further improve prediction accuracy, suggesting that the 8M model has already reached near-ceiling performance for this task in both the pre-trained and SFT settings.

Next, to investigate whether the SFT model extracts sequence patterns related to thermostability, we performed t-SNE visualization of the embedding representations from the final layer (layer 6) of the ESM-2 encoder (Fig. 3c). While the embedding space of the pre-trained ESM-2 8M model appeared to show some local clustering of high-*T_m_* sequences in certain regions, it did not exhibit a systematic, axis-aligned correlation with *T_m_* values; in contrast, the SFT model showed embedding representations clearly correlated with *T_m_* values. Specifically, in the SFT model (originally from the same pre-trained ESM-2 8M model), a strong correlation (Pearson’s *r* = −0.846) was observed between the horizontal axis (t-SNE1) in the space and *T_m_* values, indicating that thermostability-related features are captured as representations. To assess whether this pattern depends on model size, we additionally performed the same t-SNE analysis using the ESM-2 650M pre-trained model, confirming that the larger model similarly shows no clear *T_m_*-correlated structure in its pre-trained embedding space (Fig. S2, available as supplementary data at *Bioinformatics* online). This further supports that thermostability-relevant representations are acquired through supervised fine-tuning rather than scaling models alone.

### 3.2 SAE of the SFT model disentangles important features for nanobody thermostability

Through our analysis thus far, we found that the SFT model has acquired effective embedding representations for predicting thermostability. Next, to unravel what specific features comprise these internal representations, we used SAEs to disentangle the embedding representations from the final layer (layer 6) of the ESM-2 encoder with sparse representations. We applied SAEs to both the pre-trained and SFT models for comparison to verify whether the SFT model’s SAE truly extracts features important for thermostability prediction.

First, we evaluated whether the sparse representations obtained by SAE could sufficiently approximate the ESM-2 embedding representations. We added a linear regression model predicting *T_m_* values using sparse representations as input and examined whether its prediction performance was equivalent to the original linear regression model using the embedding representations as input (Fig. 4a for the SFT model; see Fig. S3, available as supplementary data at *Bioinformatics* online for the pre-trained model). In the SFT model, the prediction accuracy from sparse representations was Spearman = 0.787, quantitatively similar to the prediction accuracy from the original dense embedding representations (Spearman = 0.780). Furthermore, the correlation of predicted values was extremely high at Pearson = 0.993 (Fig. 4a), indicating that the sparse representations almost completely approximate the information related to *T_m_* prediction. Similarly, in the pre-trained model, the *T_m_* prediction accuracy from sparse representations showed Spearman = 0.739 and high correlation with predictions from the original embeddings (Pearson correlation = 0.890) (Fig. S3, available as supplementary data at *Bioinformatics* online). The slightly lower reconstruction fidelity of the pre-trained model compared to the SFT model can be attributed to the fact that fine-tuning consolidates the embedding space around a smaller number of task-relevant directions, making it more amenable to sparse approximation under the same L1 regularization constraint.

**Figure 4:**
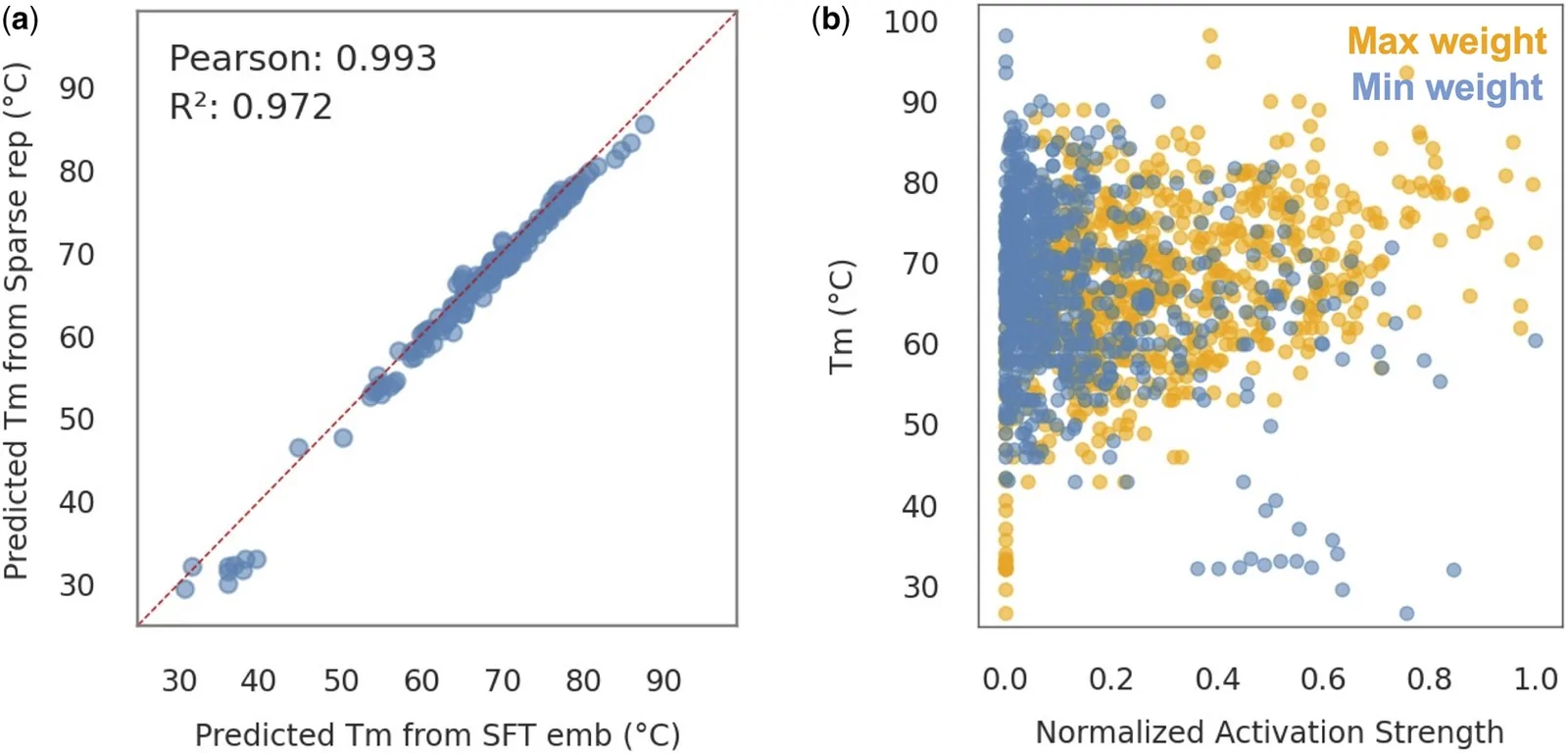
Sparse features from the SFT model capture nanobody thermostability. (a) The *T_m_* values predicted from the sparse representation of the SFT model are highly correlated with those predicted from the original dense embedding, indicating that the SAE preserves the predictive information. (b) The activation strengths of the most important positive (maximum weight) and negative (minimum weight) features of the SFT model against the true *T_m_* values.

Next, to investigate whether the SFT model’s sparse representations truly succeed in disentangling features related to thermostability, we examined the relationship between feature activation strengths (in the sparse representation) and *T_m_* values for components with large weights in the linear model (Fig. 4b). Specifically, we identified the maximum and minimum weight components in the linear model and examined the relationship between activation strength (the elements of $z_{\mathrm{pool}}$ in Fig. 2) and *T_m_* values. In the SFT model, the feature with the maximum positive weight showed good correlation between *T_m_* and activation strength, while the feature with the most negative weight showed good negative correlation between *T_m_* and activation strength (Fig. 4b and Fig. S5, available as supplementary data at *Bioinformatics* online). This indicates that the sparse representations disentangled from the SFT model retain important features for nanobody thermostability and instability. In contrast, the pre-trained model showed no clear correlation like that seen in the SFT model (Fig. S6, available as supplementary data at *Bioinformatics* online). This suggests that the pre-trained model’s ESM-2 encoder does not acquire biophysical concepts specialized for *T_m_*, having embedding representations mixed with various protein properties learned through the masked language model task.

### 3.3 Global pattern interpretation of SAE features

We visualized how SAE features globally contribute to nanobody stability across all sequences. For all sequences in NbBench thermo-seq dataset, we aggregated the activation value at each AHo numbering position weighted by the linear probe model and obtained the averaged activations at each position over the sequences. This averaged profile was separately calculated for positive weights (stabilizing) and negative weights (destabilizing), respectively (Fig. 5a). We further mapped these activations onto AlphaFold 3 predicted structures of the sequences with the highest and lowest *T_m_* in the dataset to examine their spatial distribution (Fig. 5b).

**Figure 5:**
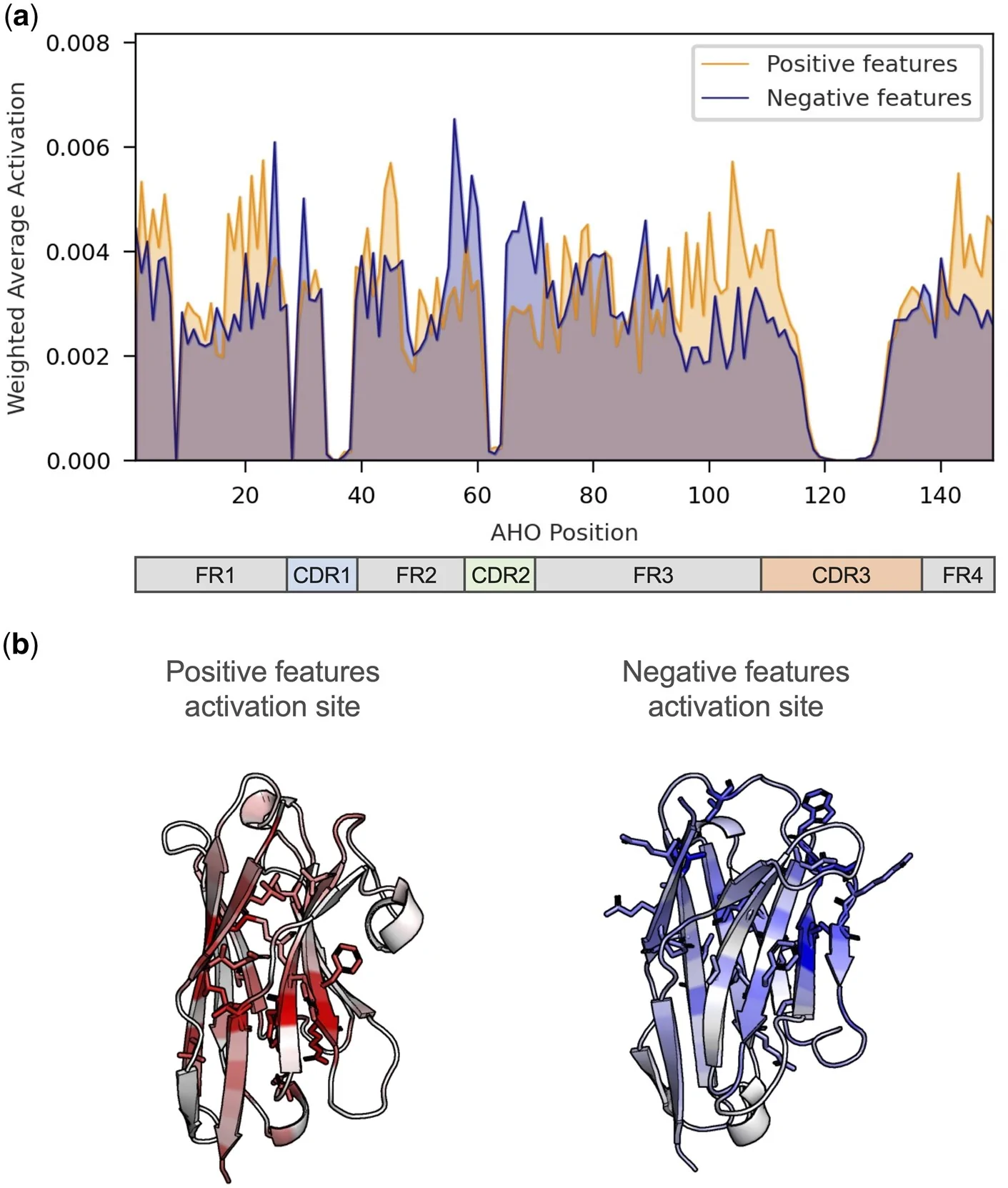
Global mapping of SAE-derived feature contributions to nanobody stability. (a) AHo-aligned, position-dependent distributions of averaged activation, shown separately for stabilizing (positive-weight) and destabilizing (negative-weight) feature sets. Contributions from both feature sets are distributed across framework and CDR positions, while the central portions of the flexible CDR loops show comparatively low averaged contributions. (b) Structural views of the same contributions mapped onto AlphaFold 3 structural models of the highest-*T_m_* and lowest-*T_m_* sequences. These aggregate maps are presented descriptively and are not used to infer a predominant region-specific mechanism.

Figure 5 shows position-dependent contributions distributed across both framework and CDR regions for both positive- and negative-weight features. Although negative-weight contributions are present around CDR2, comparable contributions are also observed at several framework positions; we therefore do not interpret destabilizing features as being predominantly associated with CDR2. The clearest aggregate pattern is the comparatively low contribution in the central portions of the flexible CDR loops. Because this position-averaged pattern was sensitive to model perturbation, we treat Fig. 5 as a descriptive summary rather than evidence for a region-specific mechanism. Residue-specific mechanistic interpretation is therefore restricted to the individual features analyzed in Section 3.4 and supported by the FEP calculations.

### 3.4 Local pattern interpretation of SAE features

We then analyzed local amino acid patterns identified by the SAE and mapped them onto nanobody model structures (Fig. 6). First, we examined amino acid residues firing in features with large positive weights of the linear model. As already shown in Fig. 4b, residues firing in these features contribute to improving *T_m_* values. In the following, all residue positions are reported using AHo numbering (Honegger and Plückthun 2001), a structure-based canonical numbering scheme for immunoglobulin variable domains that enables consistent position comparison across different nanobody sequences.

**Figure 6:**
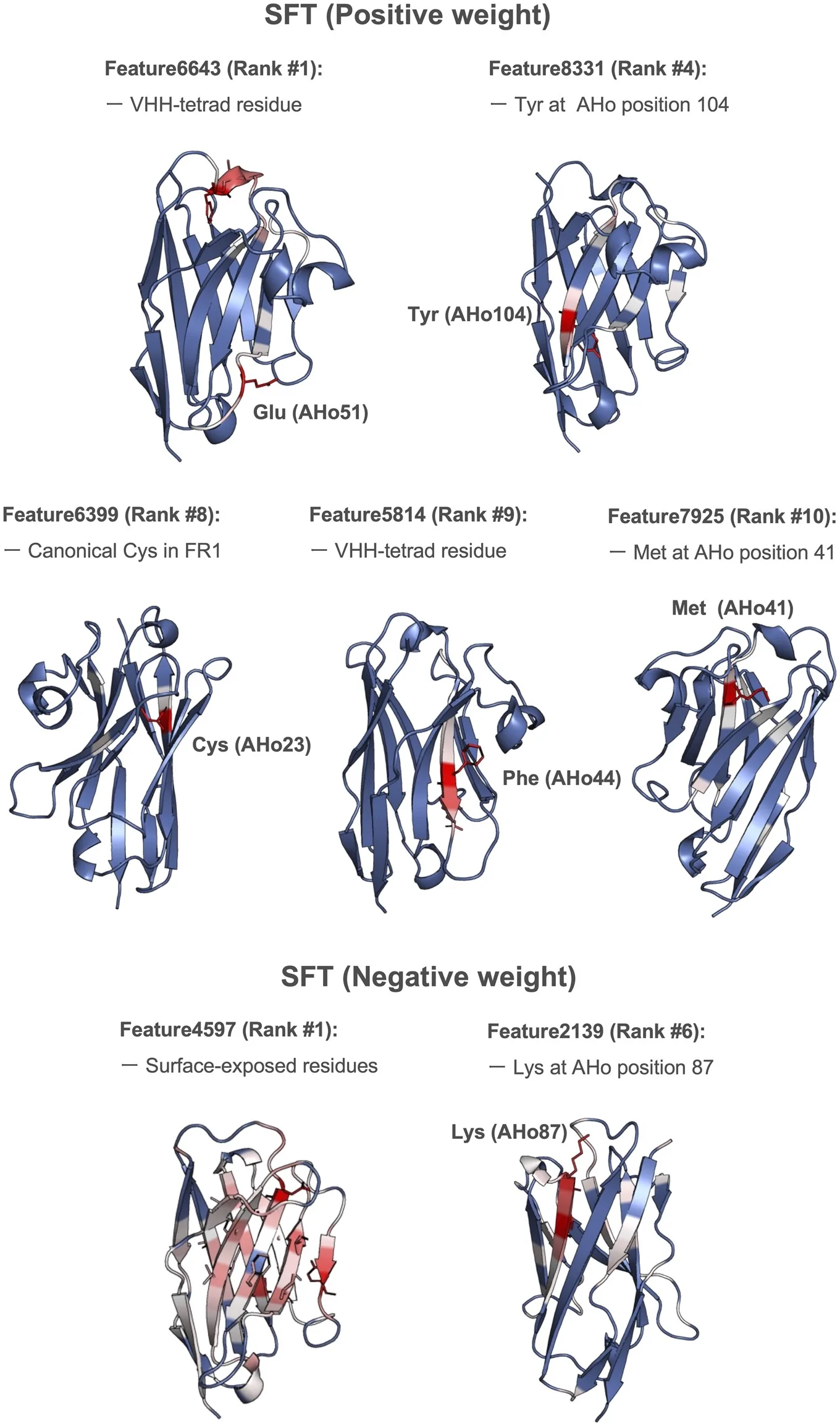
Structural interpretation of key features identified by the SAE in the SFT model. Residues highly activated by specific features are mapped onto a representative nanobody structure; full mechanistic interpretation is given in the Results text. (SFT, Positive weight) Feature 6643: Glu at AHo 51 (VHH-tetrad position); Feature 6399: Cys at AHo 23 (canonical FR1–FR3 disulfide cysteine); Feature 5814: Phe at AHo 44 (VHH-tetrad position, FR2); Feature 7925: Met at AHo 41 (FR2 hydrophobic-core residue; FEP-validated, Table 1); Feature 8331: Tyr at AHo 104 (FR3–CDR3 junction hydrophobic core; FEP-validated, Table 1). (SFT, Negative weight) Feature 4597: surface-exposed residues on the _**β**_-sheet scaffold; Feature 2139: Lys at AHo 87 (charged FR3 surface residue).

For the feature with the largest positive weight (Feature 6643), identifying residues in the highest-*T_m_* sequence revealed that Glu at AHo 51 showed strong activation. Glu at AHo 51 is one of the VHH-tetrad positions (H44, H51, H52, H54), which are hallmark substitutions where hydrophobic residues at the VH/VL interface in conventional antibodies are replaced with hydrophilic residues in nanobodies (Muyldermans *et al*. 1994, Muyldermans 2021). This activation pattern was consistently observed in other high-*T_m_* sequences as well (Fig. S12, available as supplementary data at *Bioinformatics* online). These positions are known to be biophysically crucial residue groups for stabilizing nanobodies as single-domain antibodies (Rouet *et al*. 2015).

Feature 6399 (the 8th largest positive weight) showed strong activation at Cys at AHo 23, the FR1 cysteine that forms the canonical disulfide bond with its FR3/CDR3 partner cysteine, critical for maintaining nanobody structural integrity (Liu *et al*. 2019). Feature 5814 (the 9th largest positive weight) activated strongly at Phe at AHo 44, another VHH-tetrad position in FR2. This residue participates in the conserved framework packing characteristic of the single-domain antibody fold, supporting hydrophobic core organization in the absence of the VL domain. Feature 8331 (the 4th largest positive weight) showed strong activation at Tyr at AHo 104, a residue at the FR3–CDR3 junction. The side chain of Tyr at AHo 104 is oriented toward the molecular interior, where its aromatic bulk contributes to the formation of a rigid hydrophobic core at the base of CDR3. We hypothesize that this interior packing stabilizes the CDR3 loop architecture by constraining conformational freedom at the loop base. Feature 7925 (the 10th largest positive weight) showed strong activation at Met at AHo 41, a hydrophobic residue in FR2. We hypothesize that Met at AHo 41 contributes to stability by optimally filling a hydrophobic pocket in the _**β**_-sandwich core of the VHH domain.

Next, we examined amino acid residues firing in features with large negative weights of the linear probe model. Feature 4597 (the largest negative weight) activated surface-exposed residues on the _**β**_-sheet core of the nanobody framework. Since solvent-exposed residues are generally expected to be hydrophilic for thermostability, activation at these positions may reflect insufficient hydrophilicity at surface-exposed sites of the _**β**_-sheet scaffold, correlating with reduced thermostability. Feature 2139 (the 6th largest negative weight) exhibited localized activation at Lys at AHo 87, a charged surface residue in FR3. The presence of lysine at this position may reflect an unfavorable local electrostatic environment or a disrupted hydrogen-bond network in this region, correlating with lower thermostability in the sequence lineages where it appears.

To test these hypotheses and validate the biophysical significance of the SAE-extracted features, we performed FEP calculations for nine point mutations across three nanobodies, spanning both stabilizing and destabilizing directions (Table 1; a negative **ΔΔ***G* indicates a stabilizing mutation). For Feature 8331 and Feature 7925, the SAE-identified target positions yielded the most favorable **ΔΔ***G* values among the three positions tested for each substitution (AHo 104 for Tyr and AHo 41 for Met; Fig. S11, available as supplementary data at *Bioinformatics* online). We additionally tested two SAE-highlighted positions in the destabilizing direction, using the highest-*T_m_* sequence in the dataset (seq1) as background: disrupting the canonical disulfide-forming Cys at AHo 23 (Feature 6399, discussed above) and introducing Gly at AHo 141 (flagged by a negative-weight feature) were both destabilizing by FEP, consistent with the SAE’s assignments. For the nine mutations, the stability direction inferred from **ΔΔ***G* was agreed with the SAE probe’s predicted **Δ***T_m_* in 8 of 9 cases. Overall, the FEP calculations support the ability of the SAE to prioritize context-dependent positions at which specific residues are energetically favorable or disruptive to the local structural environment of the nanobody scaffold.

**Table 1:** FEP validation results for SAE-identified positions.

Nanobody	AHo position	Mutation	ΔΔ*G*_FEP_ (kcal mol^−^**^1^)**	Δ*Tm*_pred_ (_°C_)
seq274	102	A→Y	+2.11	−0.75
	103	V→Y	+0.13	−0.22
	104	F→Y	−1.65	+0.58
seq594	40	H→M	−7.69	+0.80
	41	H→M	−8.89	+1.11
	42	G→M	−2.66	+2.07
seq1	141	Q→G	+0.66	−2.18
	22	S→A	−0.46	−1.69
	23	C→A	+1.40	−17.57

Free-energy perturbation (FEP) validation of nine SAE-identified point mutations across three nanobodies (seq274, seq594, seq1), spanning both stabilizing and destabilizing directions (positive ΔΔ*G_FEP_* = destabilizing). For each mutation, the FEP-computed free-energy change (ΔΔ*G_FEP_*) is listed alongside the SAE probe's predicted *T_m_* change (Δ*Tm_pred_*) for reference; the direction of the stability change inferred from ΔΔG was consistent with that indicated by Δ*Tm_pred_* in 8 of the 9 mutations.

Apart from these, several of the remaining top-10 positive-weight features and top-10 negative-weight features lacked spatially localized activating residues and were difficult to interpret (Fig. S7, available as supplementary data at *Bioinformatics* online).

To assess whether these localized activation patterns reflect generalizable biophysical signal rather than similarity to specific training sequences, we compared feature activation profiles between low- and high-sequence-similarity subsets of the test set (≤80% vs. >80% maximum identity to the training set) for the top 10 positive-weight and top 10 negative-weight features, and found them to be highly correlated (mean Pearson *r* = 0.81 across all 20 features; Figs S9, and S10, available as supplementary data at *Bioinformatics* online).

In contrast to the SFT model, features identified in the pre-trained model were qualitatively different from those captured by the SFT model, with consistently localized activation (Fig. S8, available as supplementary data at *Bioinformatics* online). As shown in Fig. S6, available as supplementary data at *Bioinformatics* online, these pre-trained features do not correlate with nanobody thermostability.

Collectively, these findings highlight two useful capabilities of the fine-tuned PLM. First, the model prioritizes positions at which particular substitutions are energetically more favorable than substitutions at neighboring positions, consistent with a position-specific local structural context. Second, the model proposes non-native, potentially stability-enhancing substitutions that were not observed in the examined sequences. Together, these results demonstrate the potential of the SAE-based approach to generate testable hypotheses for rational nanobody stabilization.

## 4 Conclusions

In this study, we have demonstrated that a combination of SFT and SAE serves as an effective computational protocol for mechanistic interpretability of task-specific PLMs. Here, by applying SAEs to the embeddings of this SFT model, we decomposed them into interpretable sparse representations without losing predictive performance, capturing features related to thermostability. Feature analysis revealed that features correlating with high *T_m_* values capture not only known factors like VHH-tetrad residues and disulfide bonds but also previously uncharacterized candidate stabilizing residues, while some features correlating with low *T_m_* values showed activation at surface-exposed framework residues. This approach provides a new pathway for mechanistically elucidating internal representations acquired by PLMs for specific biophysical properties, contributing to further performance improvements and rational protein design applications in the future.

While the fine-tuned PLM identifies important residues, interpretation methods require further sophistication. Currently, identifying the biophysical meaning of individual features extracted by SAEs still heavily relies on manual expert analysis, as done in this study. To accelerate and more comprehensively perform this interpretation, developing interactive visualization systems like InterPLM (Simon and Zou 2025) would be effective. Future approaches could employ large language models to automatically annotate the function and structural roles from patterns of amino acid residues. However, ensuring reliability requires verification through energy calculations using MD simulations.

The insights gained from this study lead to promising future prospects. A key direction is extending the scope of this research beyond thermostability to other biochemical or biophysical data important in antibody-based drug development, such as aggregation propensity and binding affinity to other molecules. This would deepen our understanding of universal or property-specific sequence motifs governing diverse protein properties.

## Supplementary Material

btag685_Supplementary_Data

## Data Availability

The data supporting the findings of this study have been deposited in the Zenodo repository (DOI: 10.5281/zenodo.18012026). The code is publicly available on GitHub (https://github.com/matsunagalab/paper_nanobody-thermostability-sae). Our implementation builds upon the sparse autoencoder (SAE) framework of Simon and Zou (2025) (see their original code at https://github.com/ElanaPearl/InterPLM) and incorporates only minor rather than major structural changes.
