# Supplementary material for: Mechanistic interpretability of fine-tuned protein language models for nanobody thermostability prediction
Source: Bioinformatics. 2026 Sep 19;42(10):btag685. doi: 10.1093/bioinformatics/btag685 (PMC13637583; doi:10.1093/bioinformatics/btag685)
Supplement: Supplementary file 1 [file btag685_supplementary_data.pdf]

Supplementary Data:

Mechanistic Interpretability of Fine-Tuned Protein Language Models for Nanobody Thermostability Prediction

Taihei Murakami[2,3], Yuki Hashidate[2] and Yasuhiro Matsunaga[1,2,*]

[1]Life and Medical Science Application Interface Platform Development Unit, RIKEN Center for Computational Science, Kobe, Hyogo 650-0047, Japan, [2]Graduate School of Science and Engineering, Saitama University, Saitama 338-8570, Japan, [3]Epsilon Molecular Engineering, Inc., Saitama 338-8570, Japan.

[*] ymatsunaga@riken.jp

S.1 Distribution of maximum sequence identity across NbBench thermo-seq data splits

To characterize the sequence similarity structure across data splits, we analyzed the distribution of maximum pairwise sequence identity between split pairs (test vs. train, val vs. train, and test vs. val). For each query sequence, the maximum sequence identity to any sequence in the corresponding reference split was calculated (Fig. S1). The distributions reveal that a substantial proportion of sequences share high identity (≥95%) with sequences in other splits, reflecting the sequence redundancy characteristics of the NbBench thermo-seq dataset. This pattern likely arises because the dataset is dominated by closely related nanobody variants derived from the same or similar scaffolds, and the splitting strategy prioritizes cluster-level separation over strict pairwise identity cutoffs across splits. While this structure may introduce some degree of sequence redundancy between training and evaluation sets, it also reflects the realistic distribution of experimentally characterized nanobodies, where closely related variants with measured thermostability are commonly reported together. These characteristics should be taken into account when interpreting generalization performance across splits.

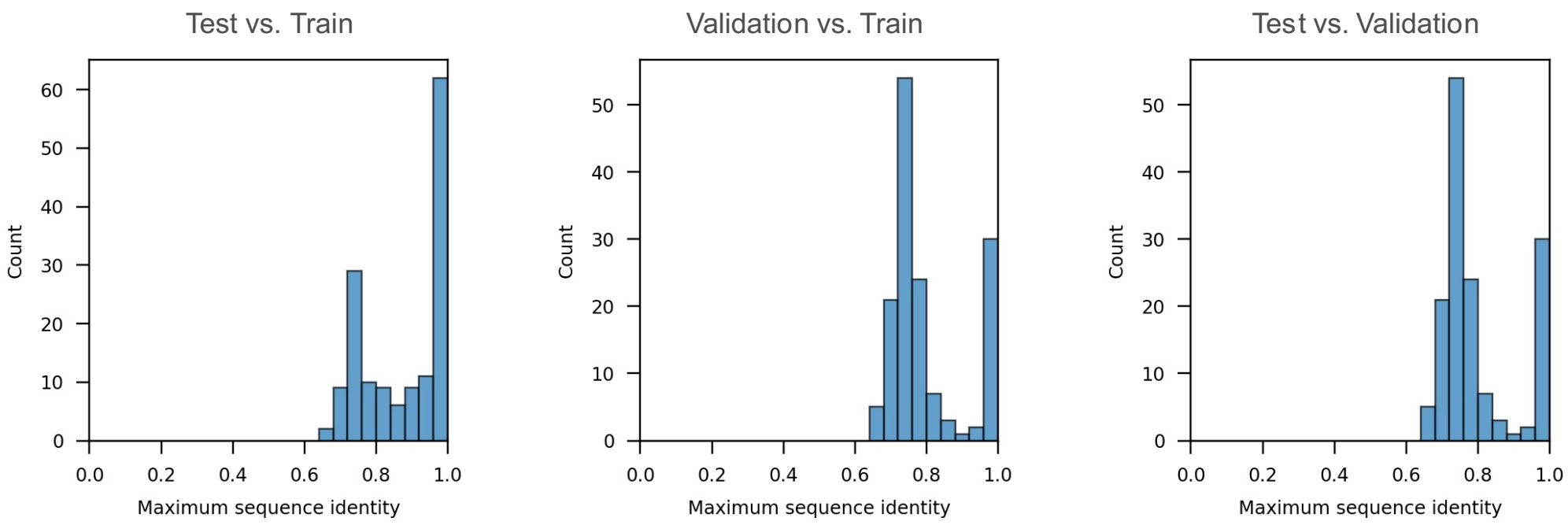

Figure S1. Distribution of maximum sequence identity across data split pairs. Histograms show the distribution of maximum pairwise sequence identity for test vs. train (left), validation vs. train (center), and test vs. validation (right) comparisons. For each query sequence in one split, the maximum sequence identity to any sequence in the reference split was calculated.

S.2 t-SNE analysis of embedding representations using thermostability-predictive dimensions

In the main text (Fig. 3c), we visualized the embedding space of the pre-trained model using t-SNE with all embedding dimensions. However, since the full embedding space captures diverse biological attributes beyond thermostability, correlations with Tm may be obscured by dominant variance unrelated to the task. To focus the analysis on dimensions most relevant for Tm prediction, we extracted the top 10 embedding dimensions with the largest absolute weights in the ridge regression prediction head and performed t-SNE using only those selected dimensions. This analysis was conducted for both the 8M and 650M parameter versions of the pre-trained and SFT models to additionally examine the effect of model size on the representational structure of thermostability-relevant dimensions.

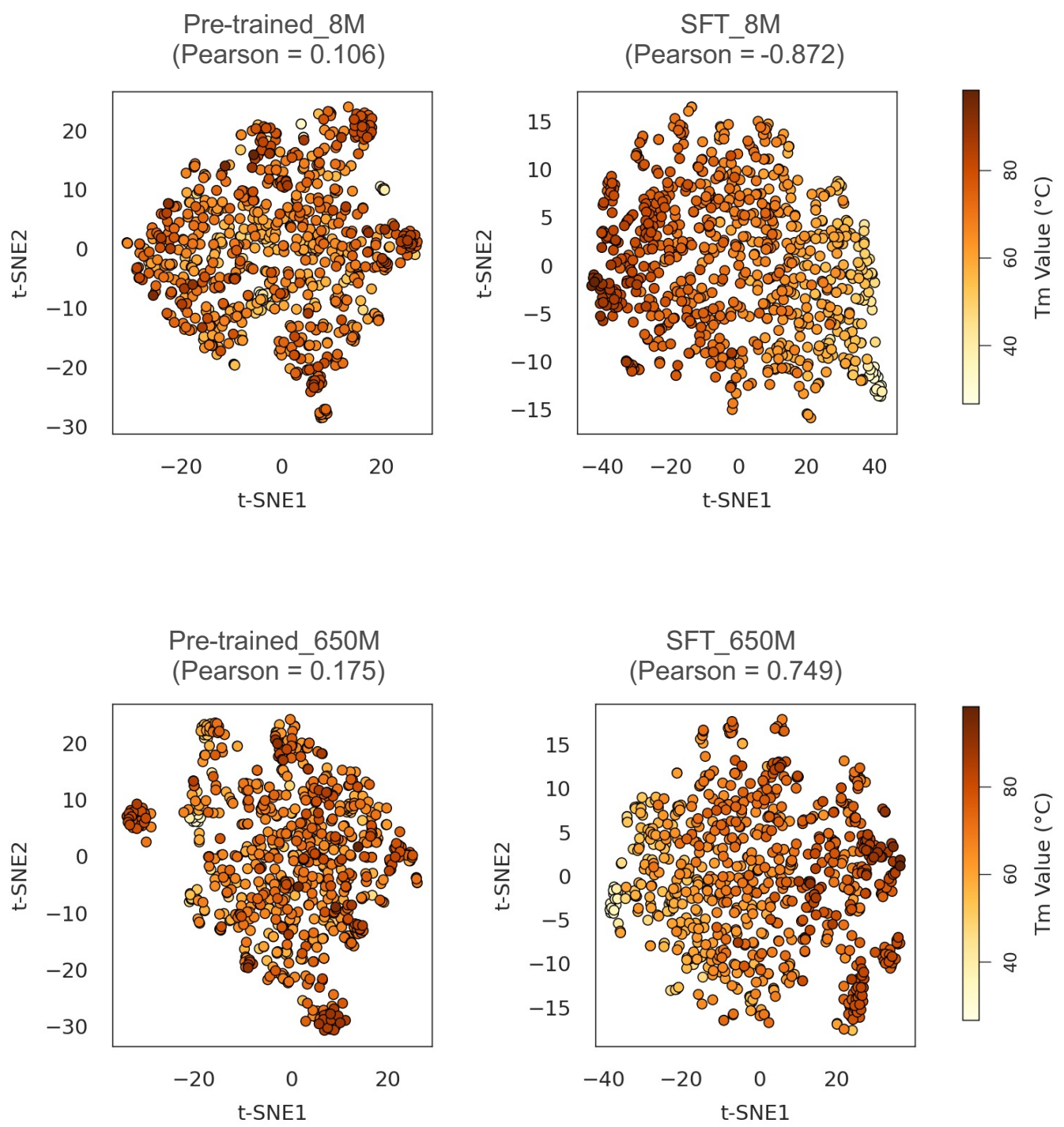

Figure S2. t-SNE analysis of pre-trained and SFT embeddings using the most relevant dimensions for Tm prediction. t-SNE projections computed from the top 10 embedding dimensions with the largest absolute weights in the ridge regression head, for the pre-trained ESM-2 8M, SFT ESM-2 8M, pre-trained ESM-2 650M, and SFT ESM-2 650M models. Each point represents a nanobody sequence and is colored by its melting temperature (Tm). The Pearson correlation coefficient between the first t-SNE component and Tm is indicated for each panel.

S.3 Nanobody thermostability prediction using sparse representations

In the main text (Fig. 4), we evaluated whether the SAE-derived sparse representations sufficiently preserve the information necessary for thermostability prediction. For the SFT model, we showed that predictions from sparse representations were highly correlated with those from the original dense embeddings (Fig. 4a), while Fig. 4b shows the relationship between the activation strengths of the maximum- and minimum-weight features and the true Tm values. Here, we provide predicted-versus-true Tm plots for both the pre-trained and SFT sparse representations, together with the agreement between sparse-based and dense-embedding-based predictions for the pre-trained model.

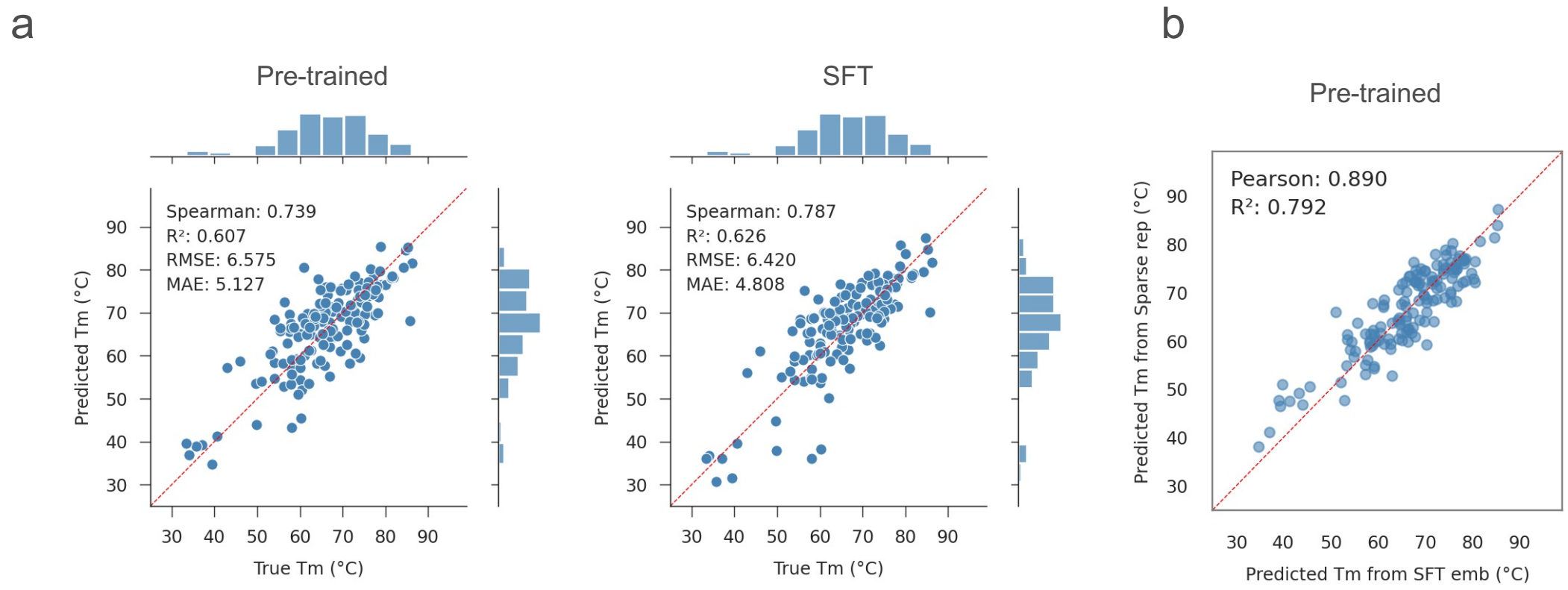

Figure S3. Nanobody thermostability prediction from sparse representations of the pre-trained and SFT models. a. Scatter plots of predicted versus true melting temperature (Tm) for the pre-trained ESM-2 model (left) and the SFT model (right), where predictions were obtained using SAE-derived sparse representations as input to the ridge regression head, evaluated on the test dataset. Each point represents a nanobody sequence. The Pearson correlation coefficient (r) and coefficient of determination (R^2) are indicated in each panel. **b.** Scatter plot comparing Tm values predicted from the SAE-derived sparse representation against those predicted from the original dense embedding of the pre-trained model, evaluated on the test dataset. The corresponding result for the SFT model is shown in Fig. 4a of the main text.

S.4 Feature ablation analysis of the SAE-based Tm predictor

To assess how predictive information is distributed across the 10,240-dimensional sparse representation, we performed a feature-ablation analysis on the held-out thermo-seq test set (*n*=147). SAE latents were ranked by the absolute value of their ridge regression weight |*w*| and progressively set to zero, with the same number of randomly selected latents used as a control. Random ablation was repeated for 1,000 draws. The 95% confidence intervals were computed using 1,000 percentile-bootstrap resamples of the test set; for random ablation, latent selection was resampled jointly with the test set. Changes in the small-*N* regime, including *N*=5, 10, and 20, were small relative to bootstrap uncertainty and non-monotonic. We therefore extended the analysis to *N*=2,000 to identify the scale at which a systematic difference between targeted and random ablation emerged.

For small *N*, targeted and random ablation both remained within the uncertainty of the no-ablation baseline. Performance degraded consistently only after several hundred of the highest-weight latents had been removed, whereas random ablation remained close to baseline throughout the tested range. These results do not support the interpretation that a handful of features carry the prediction. Instead, they indicate that predictive information is preferentially distributed across a broader, partially redundant subset of high-weight features rather than being uniformly distributed across the representation.

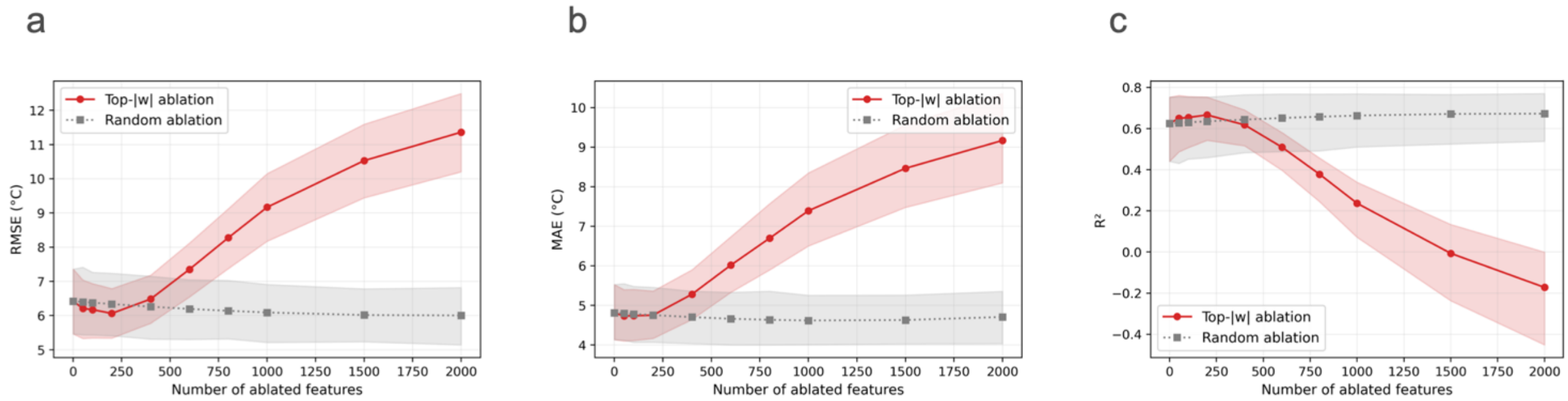

Figure S4. Feature ablation analysis of the SAE-based Tm predictor. a. RMSE, **b.** MAE, and **c.** R^2 on the thermo-seq test set (*n*=147) as a function of the number *N* of ablated SAE latents. Red: latents ablated in order of decreasing |ridge weight| (top-|*w*| ablation). Gray: the same number of latents chosen at random, averaged over 1,000 trials (random ablation). Shaded bands denote 95% confidence intervals (bootstrap percentile method, 1,000 resamples; see Supplementary Text S.4 for the top-|*w*| vs. random procedure). The broader ablation range is shown because changes at small *N* were within bootstrap uncertainty and did not support separate interpretation.

S.5 Correlation analysis between feature activation and nanobody thermostability

We presented the relationship between activation strength and Tm values only for the features with the largest positive and negative weights (Fig. 4). To verify the generality of these observations, we extended this analysis to the top 10 features with the largest positive weights and the bottom 10 features with the largest negative weights for both the SFT and pre-trained models.

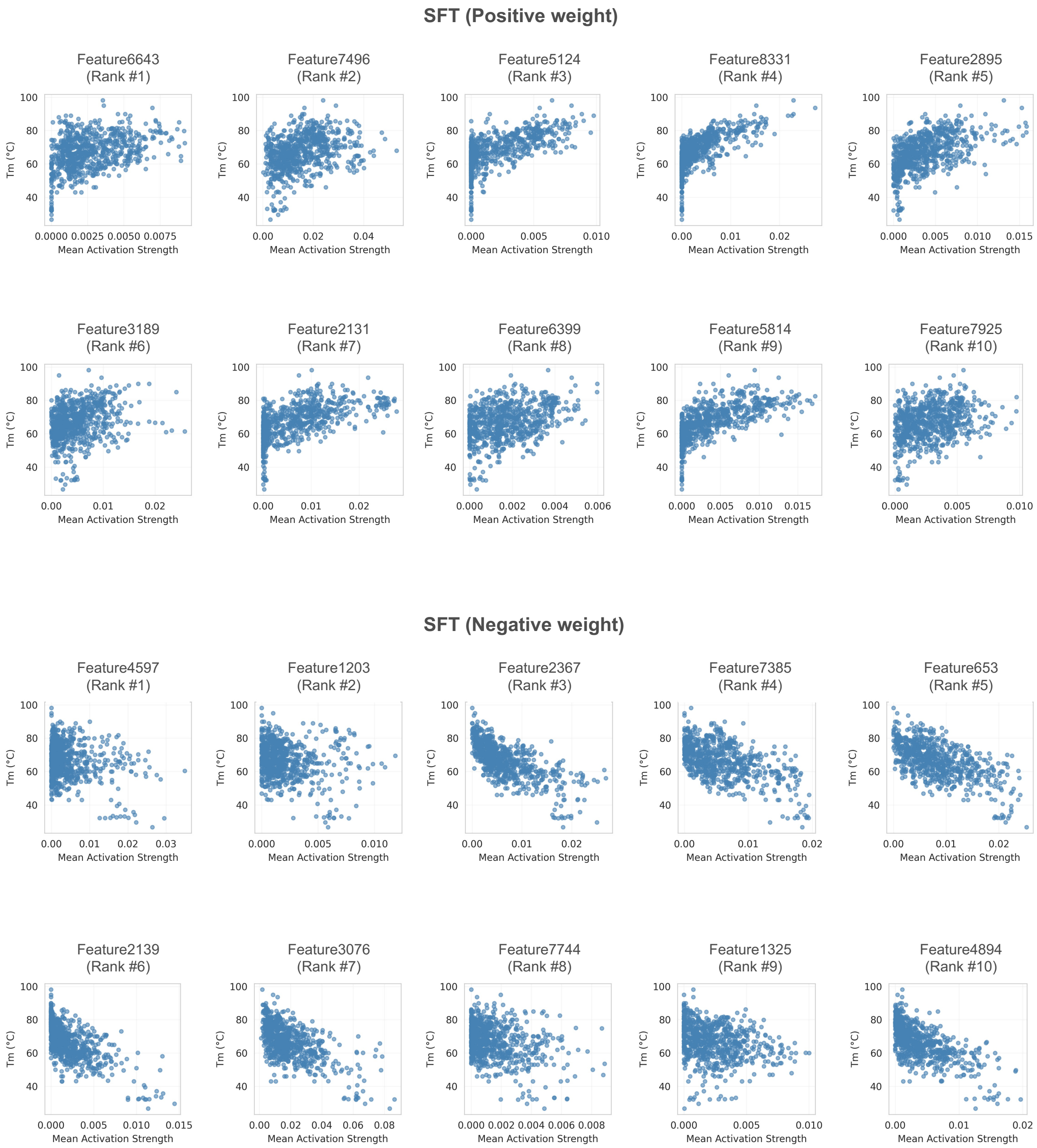

Figure S5. Relationship between feature activation and thermal stability in the SFT model. Scatter plots showing the normalized activation strength (*x*-axis) versus the true melting temperature (Tm) in the NbBench dataset (*y*-axis). (Top: Positive weight) The 10 features with the largest positive weights (Rank #1-#10) in the ridge regression head of the SFT model. (Bottom: Negative weight) The 10 features with the largest negative weights (Rank #1-#10) in the ridge regression head of the SFT model.

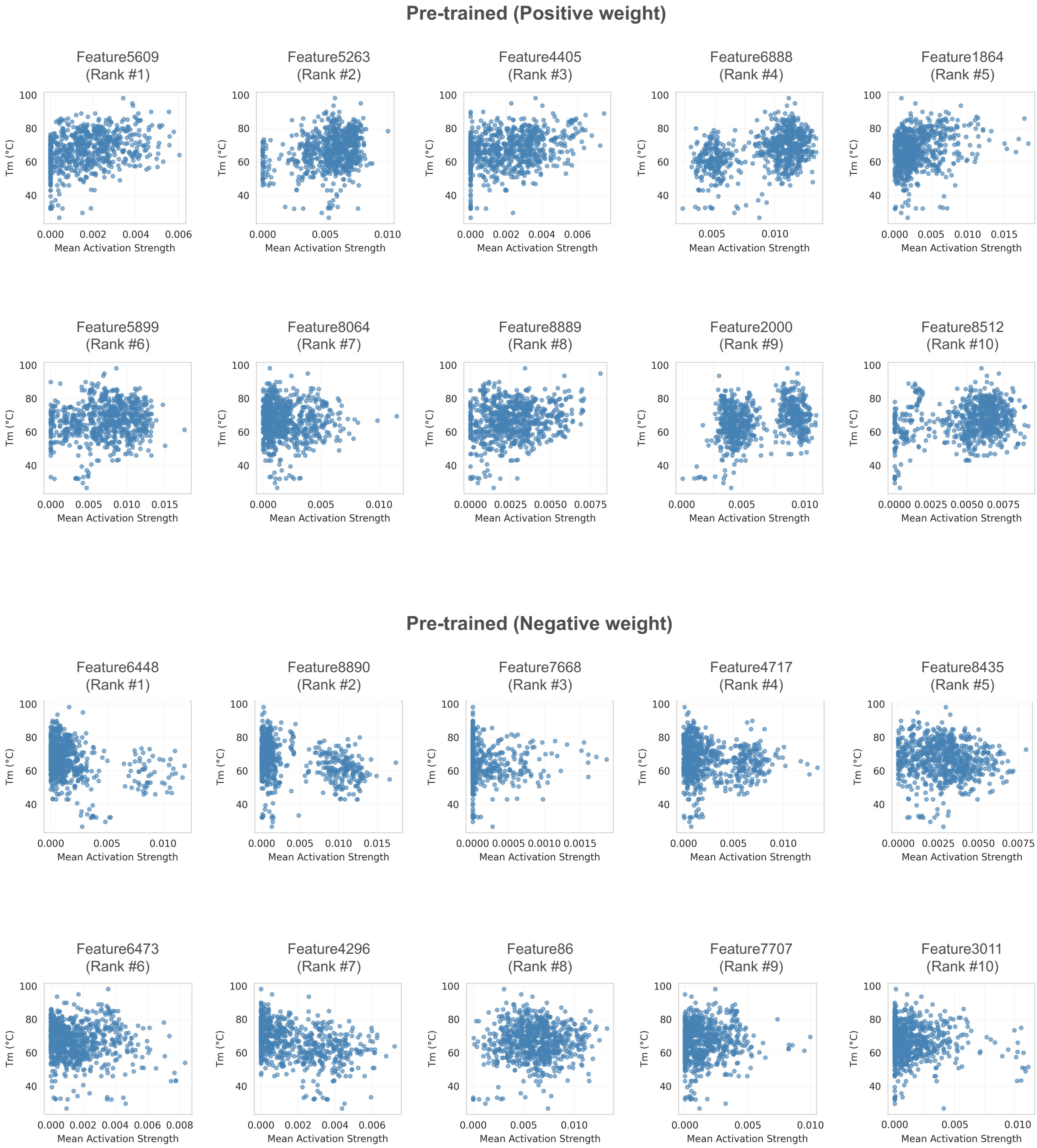

Figure S6. Relationship between feature activation and thermal stability in the pre-trained model. Scatter plots showing the normalized activation strength (*x*-axis) versus the true melting temperature (Tm) in the NbBench dataset (*y*-axis). (Top: Positive weight) The 10 features with the largest positive weights (Rank #1-#10) in the ridge regression head of the pre-trained model. (Bottom: Negative weight) The 10 features with the largest negative weights (Rank #1-#10) in the ridge regression head of the pre-trained model.

S.6 Structural mapping of the other features

For features with the largest positive or negative weights that were not shown in the main figures, we display their activation positions in nanobodies by highlighting high-activation residues and mapping them onto representative AlphaFold 3 models.

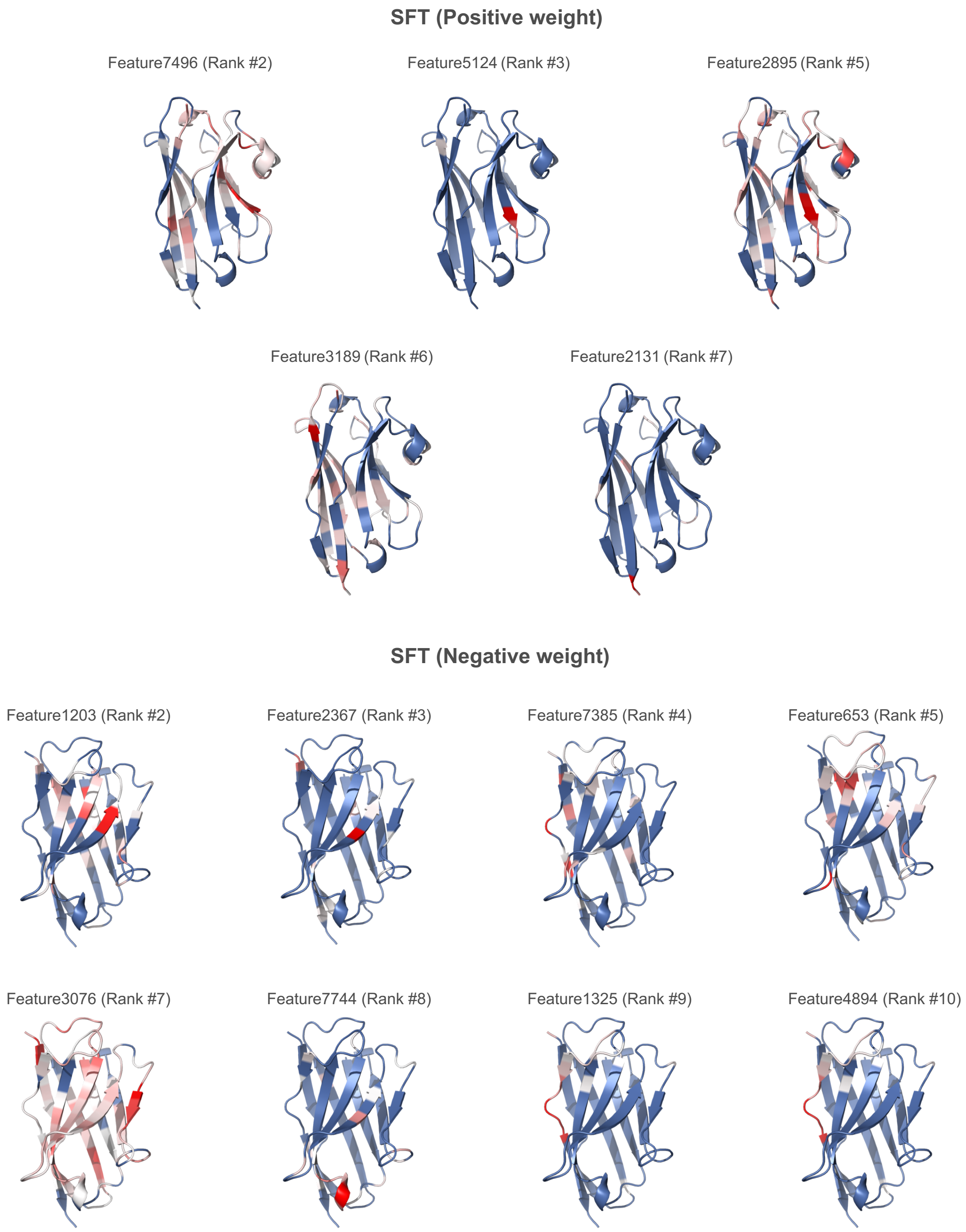

Figure S7. Structural mapping of features identified by the SAE in the SFT model. (Top: Positive weight) Positively weighted features mapped onto the model structure of the highest-Tm sequence. (Bottom: Negative weight) Negatively weighted features mapped onto the model structure of the lowest-Tm sequence.

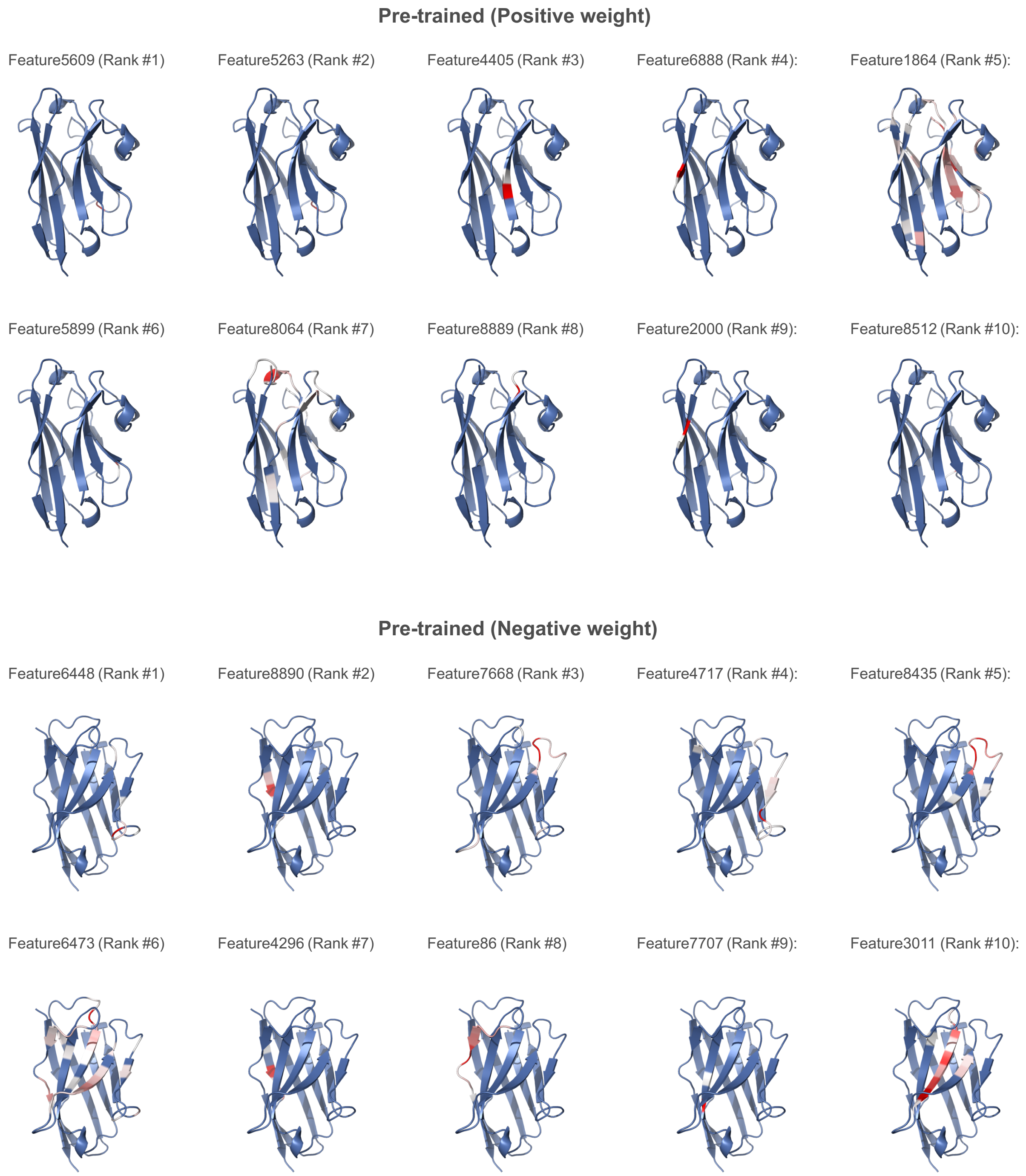

Figure S8. Structural mapping of features identified by the SAE in the pre-trained model. (Top: Positive weight) Positively weighted features mapped onto the model structure of the highest-Tm sequence. (Bottom: Negative weight) Negatively weighted features mapped onto the model structure of the lowest-Tm sequence.

S.7 Firing profile concordance for positive- and negative-weight features across low- and high-similarity subsets

To examine whether the top positive- and negative-weight SAE features fire at consistent structural positions independent of sequence-level similarity to the training set, we split the test set into a low-similarity subset (max pairwise identity to train ≤80%, n=66) and a high-similarity subset (>80%, n=81), and compared the mean per-residue (AHo-numbered) activation profile of each of the top 10 positive-weight features between the two subsets (Fig. S9; same features as Fig. S5, top). Activation profiles were highly concordant between subsets (mean Pearson r=0.90 across the 10 features), and the top-5 AHo positions of peak activation overlapped substantially (mean 3.2 of 5 positions shared), indicating that these stabilizing features activate at the same structural positions across both sequence-similarity subsets. For the bottom 10 negative-weight features, activation profiles were also concordant between subsets (mean Pearson r=0.73; mean 2.4 of 5 positions shared; Fig. S10).

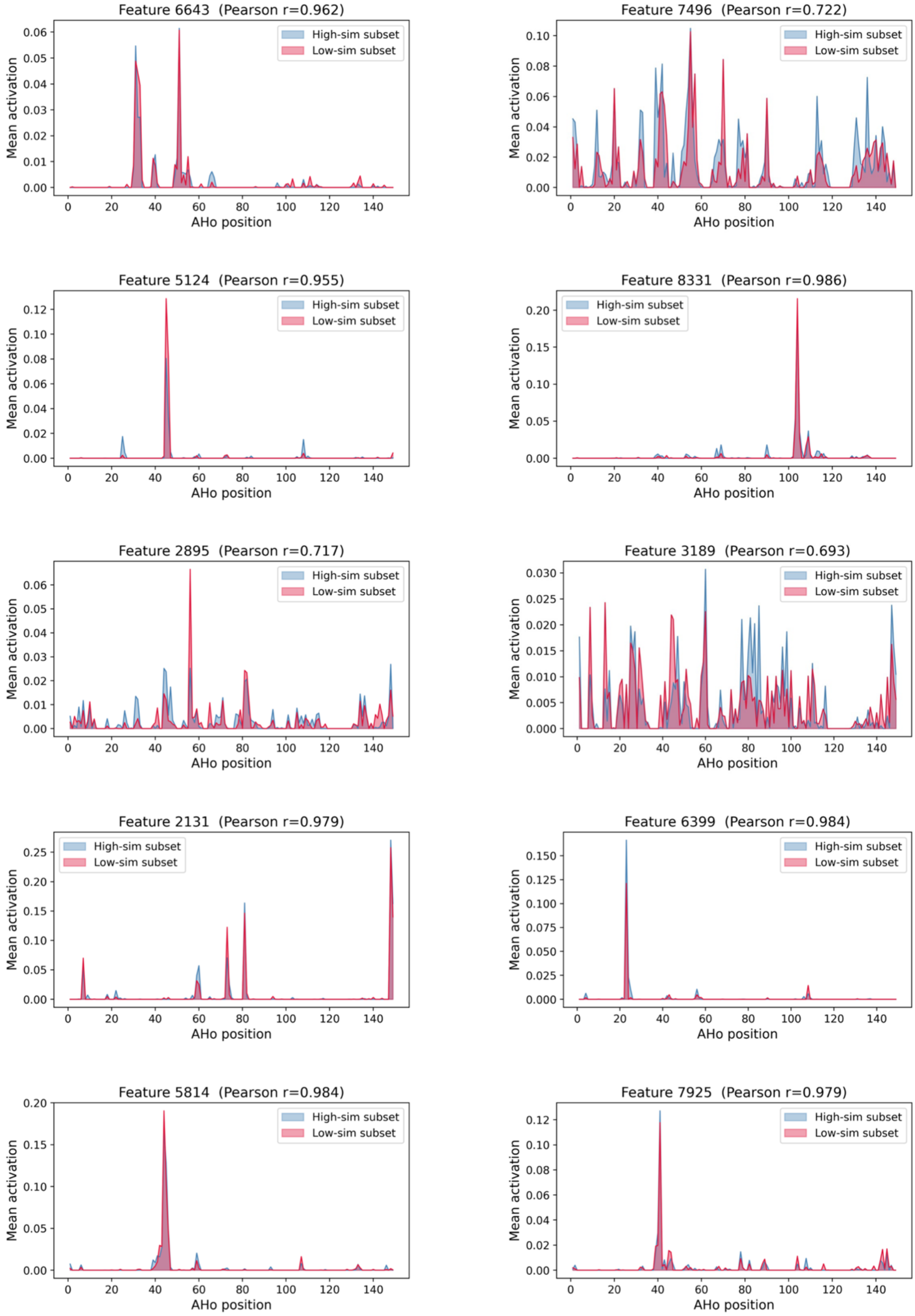

Figure S9. Firing profile comparison between low- and high-sequence-similarity subsets for the top 10 positive-weight SAE features. Each panel shows the mean activation profile across AHo-aligned positions (*x*-axis) for the low-similarity subset (red; max identity to train ≤80%, *n*=66) and the high-similarity subset (blue; >80%, *n*=81). Features are the same top 10 positive-weight features shown in Fig. S5 (top), ranked #1–#10 by ridge regression weight. The Pearson correlation coefficient (r) between the two subsets' mean profiles is indicated in each panel title.

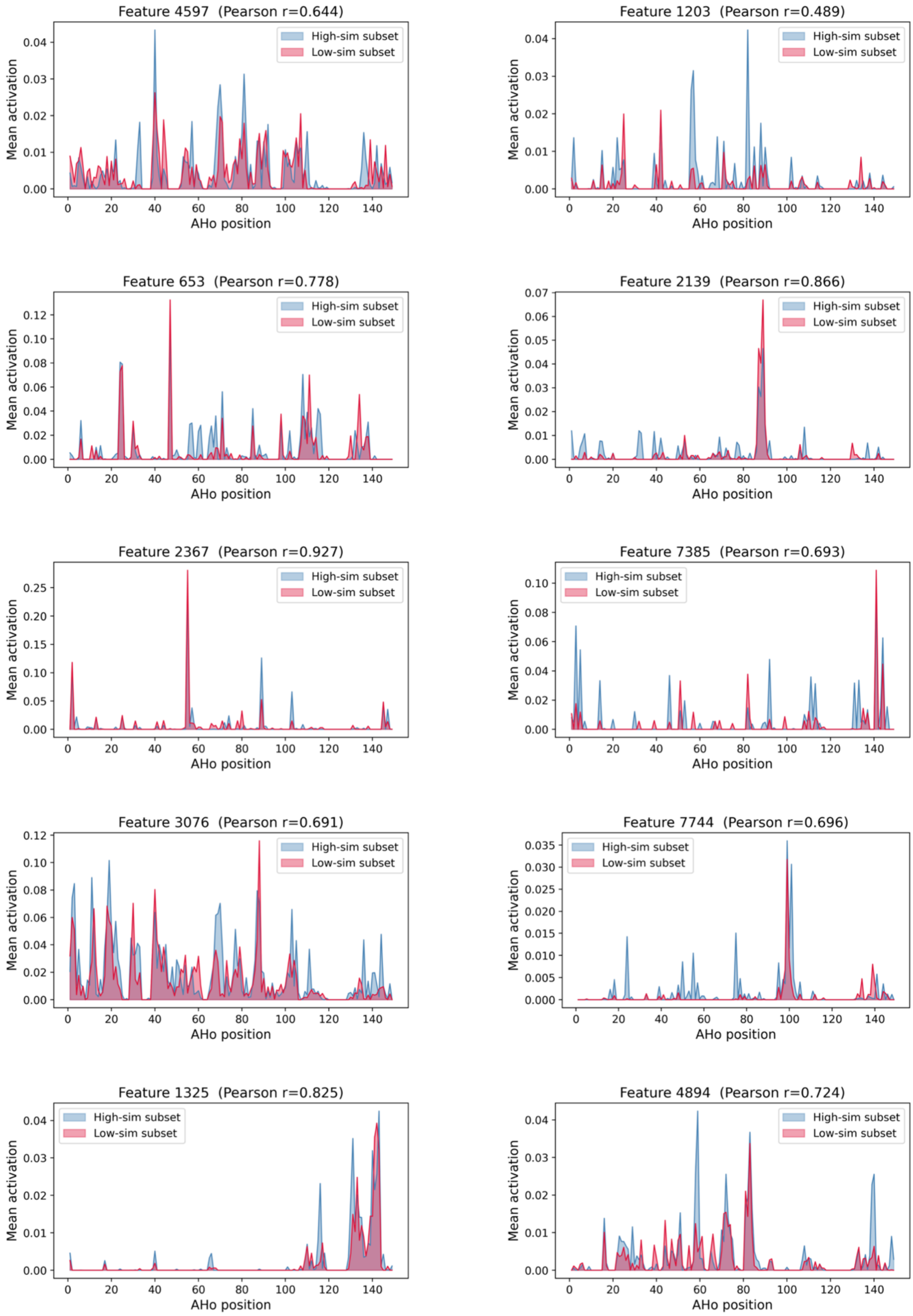

Figure S10. Firing profile comparison between low- and high-sequence-similarity subsets for the bottom 10 negative-weight SAE features. Each panel shows the mean activation profile across AHo-aligned positions (*x*-axis) for the low-similarity subset (red; max identity to train ≤80%, *n*=66) and the high-similarity subset (blue; >80%, *n*=81). Features are the same bottom 10 negative-weight features shown in Fig. S5 (bottom), ranked #1–#10 by ridge regression weight. The Pearson correlation coefficient (r) between the two subsets' mean profiles is indicated in each panel title.

S.8 Sequence and positional context of FEP calculations

For Feature 8331, FEP calculations were performed on seq274 to compare Tyr substitutions at the SAE-identified target position AHo 104 and at the neighboring positions AHo 102 (A102Y) and AHo 103 (V103Y). Among the three positions tested, the SAE-identified target position AHo 104 yielded the most favorable ΔΔG, supporting its position-specific structural relevance for Tyr. For Feature 7925, the most thermostable nanobody in the dataset (seq1, Tm = 98.23°C) carries Met at AHo 41. To compare the energetic favorability of Met at this target position with the flanking positions, FEP calculations were performed on seq594 for AHo 40 (H40M), AHo 41 (H41M), and AHo 42 (G42M). Among the three positions tested, the SAE-identified target position AHo 41 yielded the most favorable ΔΔG, supporting the biophysical relevance of the feature. We additionally performed FEP for two SAE-highlighted positions in the destabilizing direction, using seq1 as background: C23A, disrupting the canonical FR1-FR3 disulfide bond identified by Feature 6399, together with the neighboring position AHo 22 (S22A) as a flanking control, and Q141G, a substitution flagged as destabilizing by Feature 7385 (Table 1 in the main text).

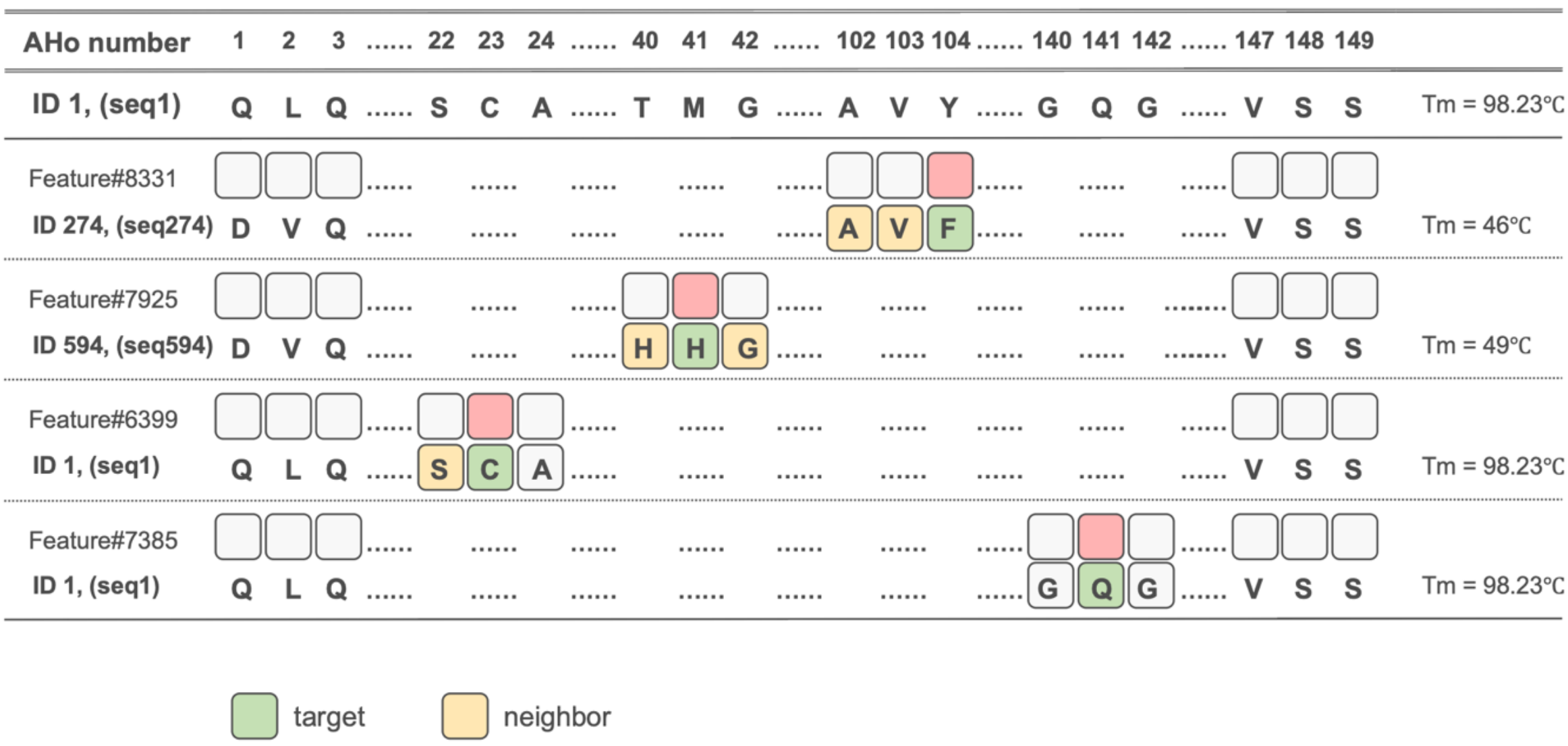

Figure S11. Representative nanobody sequences and AHo-numbered positions used for FEP validation. Each panel shows the nanobody sequence used for FEP, with AHo positions indicated; target residues (the position highlighted by the SAE feature) and tested flanking positions are distinguished by color. Feature 8331 (AHo 104, flanking AHo 102/103); test sequence seq274. Feature 7925 (AHo 41, flanking AHo 40/42); test sequence seq594. seq1 (Tm = 98.23°C, the highest-Tm sequence in the dataset) carries Met at AHo 41, motivating this substitution. Feature 6399 (AHo 23, the canonical FR1‑FR3 disulfide cysteine; flanking AHo 22) and Feature 7385 (AHo 141); test sequence seq1.

S.9 Comprehensive analysis of activation patterns for features

In the main text (Fig. 6), we mapped feature activations onto representative structures. To verify whether these activation patterns are conserved at specific positions (AHo numbering) across the entire dataset and how their intensity correlates with Tm ranking, we performed a heatmap analysis. We visualized the activations for the seven key SFT features shown in Fig. 6 (five positive-weight and two negative-weight features) across all 764 sequences sorted by Tm.

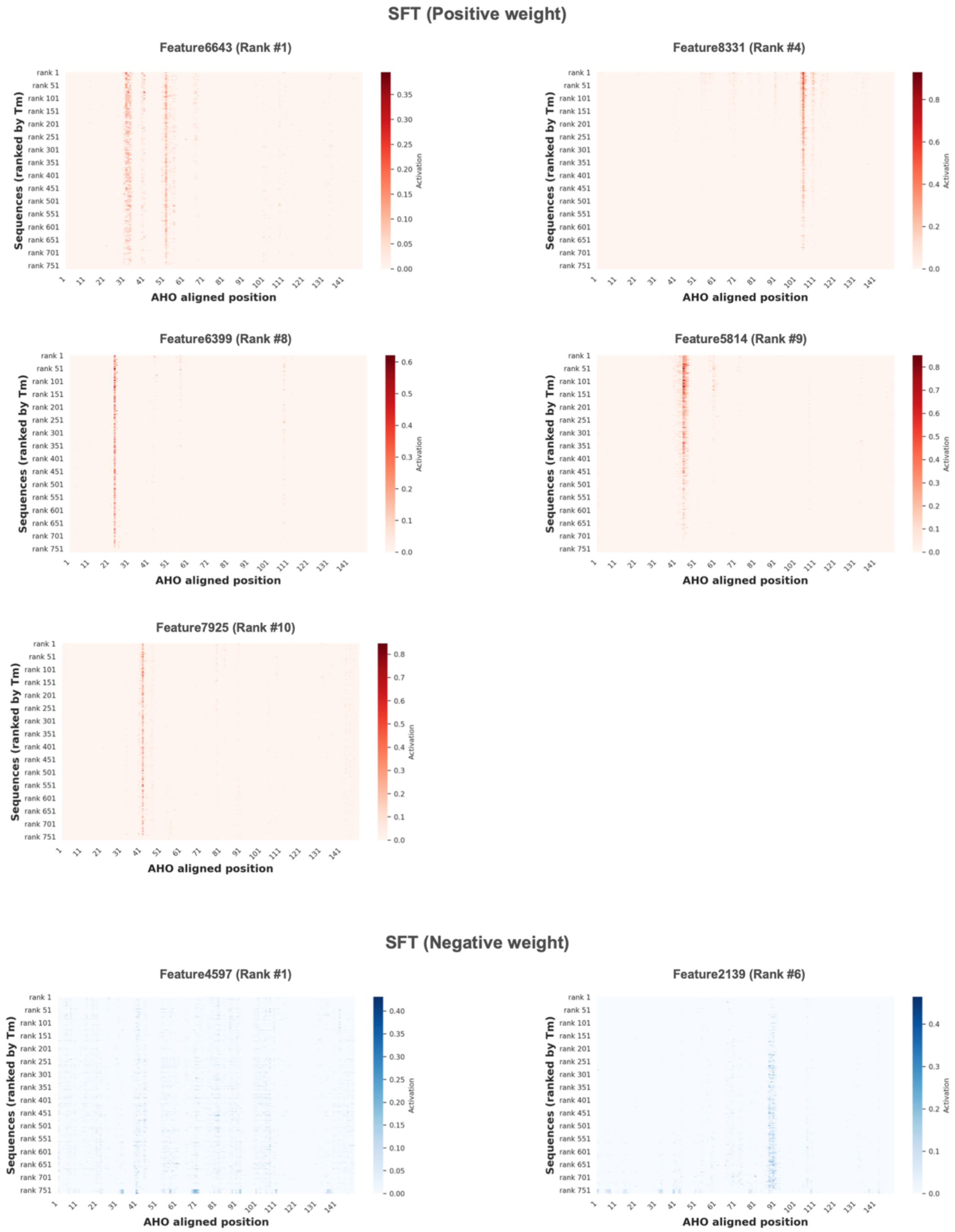

Figure S12. Heatmaps of feature activation across all AHo-aligned sequences for key features. Heatmaps showing the activation strength for the seven key SFT features shown in Fig. 6 across all 764 sequences from the NbBench thermo-seq dataset aligned via AHo numbering. The *y*-axis represents sequences sorted by Tm in descending order (Rank 1 corresponds to the highest Tm; Rank 764 to the lowest), and the *x*-axis represents the AHo-aligned positions (1‑149). Color intensity indicates normalized activation strength.
